# Individual-specific change points in circadian rest-activity rhythm and sleep in individuals tapering their antidepressant medication: an actigraphy study

**DOI:** 10.1038/s41598-023-50960-1

**Published:** 2024-01-09

**Authors:** Olga Minaeva, Evelien Schat, Eva Ceulemans, Yoram K. Kunkels, Arnout C. Smit, Marieke Wichers, Sanne H. Booij, Harriëtte Riese

**Affiliations:** 1 Department of Psychiatry (CC72), Interdisciplinary Center Psychopathology and Emotion Regulation (ICPE), University Medical Center Groningen, University of Groningen, P.O. Box 30.001, 9700 RB Groningen, The Netherlands; 2 Faculty of Psychology and Educational Sciences, KU Leuven, Leuven, Belgium; 3 Clinical Psychology, Faculty of Behavioral and Movement Sciences, VU Amsterdam, Amsterdam, The Netherlands; 4 Lentis, Center for Integrative Psychiatry, Groningen, The Netherlands

**Keywords:** Depression, Translational research, Diagnostic markers

## Abstract

Group-level studies showed associations between depressive symptoms and circadian rhythm elements, though whether these associations replicate at the within-person level remains unclear. We investigated whether changes in circadian rhythm elements (namely, rest-activity rhythm, physical activity, and sleep) occur close to depressive symptom transitions and whether there are differences in the amount and direction of circadian rhythm changes in individuals with and without transitions. We used 4 months of actigraphy data from 34 remitted individuals tapering antidepressants (20 with and 14 without depressive symptom transitions) to assess circadian rhythm variables. Within-person kernel change point analyses were used to detect change points (CPs) and their timing in circadian rhythm variables. In 69% of individuals experiencing transitions, CPs were detected near the time of the transition. No-transition participants had an average of 0.64 CPs per individual, which could not be attributed to other known events, compared to those with transitions, who averaged 1 CP per individual. The direction of change varied between individuals, although some variables showed clear patterns in one direction. Results supported the hypothesis that CPs in circadian rhythm occurred more frequently close to transitions in depression. However, a larger sample is needed to understand which circadian rhythm variables change for whom, and more single-subject research to untangle the meaning of the large individual differences.

## Introduction

Circadian rest-activity rhythm (RAR), physical activity (PA), and sleep disturbances are commonly found in individuals diagnosed with depression^1^ (see Box 1 for details on RAR, PA, and sleep parameters). Depressed individuals tend to have ﻿a delayed RAR, dampened amplitude, and lower MESOR (midline of the rhythm), indicating a more smoothed diurnal rhythm with lower daily activity levels^2,3^. Also, having more depressive symptoms ﻿is associated with lower stability and higher fragmentation of the RAR^4,5^. Others have reported lower levels of PA among depressed compared to non-depressed individuals^6^, worse reported sleep quality, and often a shorter or excessively longer sleep duration^7,8^. Longitudinal studies indicated that disturbed RAR might be found both during a depressive episode and close to the onset of depressive symptoms^9^. ﻿In a study in older men, both a late acrophase (i.e., late time of the RAR peak) alone and the combination of an early acrophase with a dampened 24-h rhythm amplitude were associated with a faster increase in depressive symptoms over 15 months^10^. Moreover, higher PA levels were protective against developing depression in older adults^11^ and the general population^12^. It can be concluded that the group-level association between depression and circadian rhythm-derived outcomes (RAR, PA, and sleep) is well-established.

Although group-level (nomothetic) studies provide valuable and relevant information about average tendencies in a subpopulation, group-level estimates often obscure relevant individual differences^13^. Variations in the strength and direction of associations (i.e., some individuals display positive associations while others have neutral or negative ones)^14^, as well as the timing of changes, can be missed in such analyses. These individual differences can be effectively explored using a repeated single-subject design, which focuses on intensive data collection at the individual level. This approach is also more in line with clinical practice in individual patients. In the present study, we adopted this approach, collecting actigraphy data in the transition in depression (TRANS-ID) Tapering study^15^, where some participants experienced a transition to a new depressive episode. Actigraphy, known for its ability to passively collect ecologically valid data on behavior, including RAR, PA, and sleep, was employed^16,17^.

**Figure 1:**
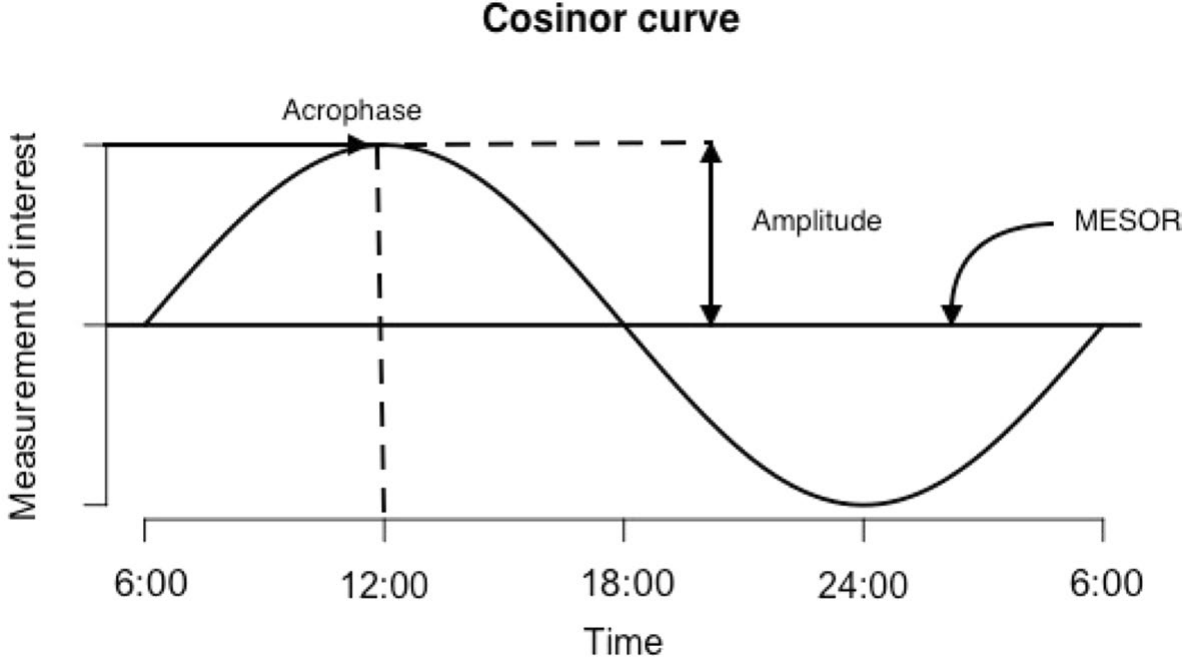
Example of a cosinor curve and terms used to describe a diurnal rhythm: MESOR, amplitude, and acrophase.

To date, it remains unexamined whether within-individual changes and disturbances in circadian rhythm dynamics are linked to the onset of depressive symptoms^18^. Hence, we adopted a single-subject approach to investigate whether elements of circadian rhythm, such as RAR, PA, and sleep, change in proximity to the emergence of depressive symptoms. Establishing this temporal link is essential, as it may shed light on whether this association plays a role in the pathophysiology or even the etiology of depression. In this study, we analyzed actigraphy data from individuals tapering their antidepressants to address three key questions: (1) Can we identify changes in RAR, PA, and sleep variables in proximity to the recurrence of depressive symptoms in these individuals? (2) Do these changes occur less frequently if participants do not develop new depressive symptoms? (3) Are there individual differences in the direction of change of circadian RAR, PA, and sleep variables similar to those observed in group-level studies? (Fig. 1).

Box. Circadian rest-activity rhythm, physical activity, and sleep parameters **Cosinor method parameters**

The cosinor method allows estimating circadian rhythms by fitting individual actigraphy data to a cosine curve of a 24-h activity rhythm and seeing the precise curve in activity level, level of activity, and occurrence of activity peak (timing) for each day

In the graph (Fig. 1), arrows indicate three main parameters that can be derived from the cosinor curve: *MESOR* (an acronym for Midline Estimated Statistic of Rhythm), which is a circadian rhythm-adjusted mean based on the parameters of a cosine function fitted to the raw data, *amplitude* is the difference between the peak and the mean value of a curve, and *acrophase* is the time at which the peak of a rhythm occurs. MESOR can also be used to evaluate daily physical activity levels

**Non-parametric circadian rhythm analysis (NPCRA) parameters**

As the RAR does not always resemble a cosine wave, it might be appropriate to apply a different method. The NPCRS is an alternative quantification method that does not require the rhythm to have a cosine-like shape to obtain valid parameters. The NPCRA includes the following parameters:

*IS* (Interdaily stability): quantifies the degree of regularity in the rest-activity pattern with a range of 0–1, where a value of 0 indicates a total lack of rhythm and a value of 1 indicates a perfectly stable rhythm

*IV* (Intra-daily variability): quantifies the degree of fragmentation of activity-rest periods. Typical healthy subjects will show a single prolonged activity period and a single prolonged rest period per 24-h cycle. The variable has a theoretical range of 0–2, with higher values indicating higher fragmentation. Typical values for healthy subjects will be below 1

*M10* (Most 10 active hours): the average activity level for the sequence of the highest (most) ten active hours. This value indicates how active and regular the wake periods are

*RA* (Relative Amplitude): the difference between the average activity level in the M10 and the L5 periods divided by the sum of the L5 and M10 to remove sensitivity to the overall activity level. The variable has a theoretical range of 0–1, with higher values indicating a rhythm with higher amplitude

**Sleep parameters**

Several sleep parameters are relevant and could be affected in depression. The most frequently used ones are *time in bed* (TIB, time duration between going to bed and getting out of bed), *sleep efficiency* (the ratio of total sleep time to time in bed), and *fragmentation index* (amount of movement or restlessness in a sleep period). The fragmentation index indicates the degree of fragmentation of the sleep period and can be used as an indication of sleep quality (or lack thereof)

## Methods

### Participants

**Figure 2:**
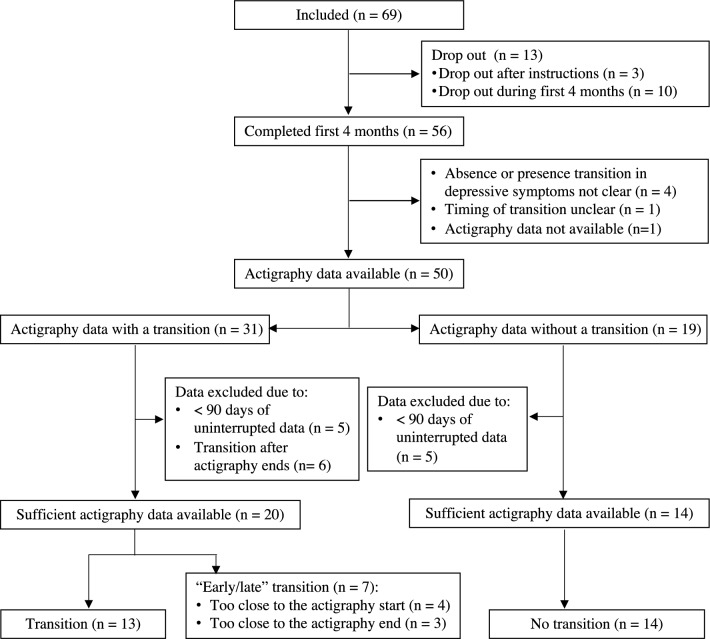
Flowchart of participants with and without a transition in depressive symptoms. *Note*. For the 56 participants who completed 4 months of monitoring, definitions for the transitions were based on the weekly SCL-90 depression subscale scores, evaluation interview, and self-reported experience of recurrence of depressive symptoms^20^. Of the 50 participants with available actigraphy data, individuals with a transition after the end of actigraphy monitoring (n = 6) and with less than 90 days of observations (n = 10) were excluded as it appeared to be insufficient for the KCP-RS analysis to reliably identify CPs in the data. With approximately 120 days of observations per person and ten variables included in the change point analysis, we expect to accurately detect (moderate and) large changes in circadian rhythm and sleep variables^47^.

The TRANS-ID sample consists of 69 remitted individuals who tapered their antidepressant medication during the study period and were, therefore, at high risk of developing depressive symptoms^19^. Participants were recruited through media advertisements, healthcare institutions, and Dutch pharmacies^15^. A number of participants were excluded from the final sample due to various reasons outlined further. A stepwise description of the inclusion process can be found in Fig. 2. Of the 34 individuals included in the final sample, 26 participants were female (76.50%), the mean age of participants was 48.56 years (range 25–67 years), 29 were married or in a relationship (85.30%), 22 had a university education (64.71%), and 23 were employed (67.65%). Two individuals worked as shift workers in the last 3 months (5.88%). Since the TRANS-ID Tapering study was strictly observational, with no interference with treatment or the tapering process, researchers did not influence the tapering process. The authors assert that all procedures contributing to this work comply with the ethical standards of the relevant national and institutional committees on human experimentation and with the Helsinki Declaration of 1975, as revised in 2008. The study was approved by the Medical Ethical Committee of the University Medical Center Groningen (UMCG, METc2016.443). All patients were informed that they could stop their participation at any time and were asked to read and provide written informed consent prior to participation.

### Study design and data collection

The study period included the last month of antidepressant tapering and the 3 months directly after that. This was done to enable capturing both the final stages of antidepressant discontinuation and the first months directly after that when one would expect depressive symptoms to return^20^. All individuals were continuously monitored for a total of 4 months with actigraphy (24 h a day, MotionWatch 8, MW8, CamNTech)^15^. The actigraphy data were recorded at a 1-min epoch length, with light detection and data compression in the MW8 disabled. Individuals were instructed to continuously wear the device, only remove it in rare situations, such as sauna visits, and press the event marker button when going to sleep and getting up^21^. In addition, participants filled out the Symptom Checklist (SCL)-90 depression subscale^22^ on their smartphone (~ 26 assessments per participant) each weekend for a period of 6 months, starting together with actigraphy assessments and continuing for two additional months. This study’s design, hypotheses, and analysis plan were preregistered before conducting, and an extended version of the methods is available on the OSF page (https://osf.io/f45qg). A few deviations from the preregistration occurred while conducting the study and were reported in Supplementary Material 1.

### Data preprocessing

There were two reasons for missing days of actigraphy data, and several pre-processing steps were done to deal with missing data. First, due to the limited battery life of MW8 (approximately 2 months), at least two MW8 devices were used for each participant, and then at least two actigraphy data files were joined into one uninterrupted time-series file. On a few occasions, the joining process resulted in several missing days around the MW8 changes (e.g., the second MW8 arrived a few days after the first MW8 stopped working). If time-series data from participants contained at least six consecutive days of missing data, it was considered interrupted and unfit for successful analysis execution. Therefore, the data were excluded if the longest part was shorter than 90 days (see more details in the “Statistical analysis” section). Second, the actigraphy data could be missing due to prolonged non-wear time. As we were interested, among others, in circadian RAR variables, we excluded the data of the whole day (24 h) if more than four consecutive hours were missing during the day (~ 16,67% of the daily data). This was done since missing a substantial part of the day would interfere with calculating RAR variables and produce biased estimates. As there is no established cut-off for excluding the missing actigraphy data when analyzing daily rhythms, we employed the cut-off used in earlier studies with a similar research focus^23,24^. Missing data for up to 4 h was imputed using the R-package MICE^25^ (for details, see Supplementary Material 2). Handling missing data was done by imputing a particular missing epoch (1 min) as the average activity counts of the same epoch over 7 days before and after the missing epoch with available data for that epoch interval. We chose to restrict the imputation interval by a 1-week period around the missing data to account for possible changes in actigraphy data due to depressive symptom transitions. This approach has been previously shown to be fairly robust for imputing actigraphy data^26^. Out of 20 individuals with the imputed data, 8 individuals had data from 1 day (up to 4 h max) imputed, 4 individuals had data from 2 days imputed, 3 individuals had data from 3 days imputed, 3 individuals had data from 4, 1 individual had data from 7 days imputed, and 1 individual had data from 10 days imputed. Neither of the imputed days had a connection with the identified CPs. For two individuals, the imputed days occurred consecutively, limited to 2 days in a row.

### Transition in depressive symptoms and change points

The outcome variable was the transition timing toward higher levels of depressive symptoms based on the criteria formulated by Smit et al.^20^. Transitions were based on quantitative (i.e., weekly SCL-90 depression subscale scores) and qualitative information i.e., (i) open comments in weekly questionnaires, (ii) emails and phone calls, and (iii) open questions in the evaluation interviews, enquiring about participants’ mood, depressive symptoms, and treatment).

There were two quantitative criteria to determine if a transition occurred: reliable change and persistent change. A change was considered reliable if depressive symptoms were 8.5 or more points higher than the average level of depressive symptoms during the first 2 weeks of the study. A persistent change required a reliable increase in the SCL-90 score to persist for at least three consecutive weeks.

Qualitative information was assessed by three independent and qualified raters (two psychologists and one psychiatrist) to evaluate the real-life experience of depressive symptoms. Raters assessed the following questions: “Did the patient experience a change in depressive complaints that had a clear and meaningful impact on the daily life of the patient” (Yes/No/Unknown), “Did the participant describe the increase in their depressive symptoms as sudden?” (Yes/No/Unknown), and “On which date did the participant first report they were suffering from an increase in depressive symptoms?”. Qualitative results were based on the final consensus rating. The presence of the quantitative symptom change (i.e., both a reliable and persistent change) and the qualitative rating were required to include a transition in this study.

The transition variable was used to examine whether changes in RAR, PA, and sleep variables occur in proximity to a transition in depressive symptoms. Those changes were based on change points (CPs) identified by the Kernel Change Point detection on the running statistics (KCP-RS) method (for details, see “Statistical analysis” section). Detected CPs were considered in proximity when they happened within 14 days before or after a transition week. Since transitions were based on weekly reports, we took the whole week rather than 1 day to define a moment of transition. To assess the proximity of the identified CP to a transition, we used the first and the last day of the transition week ±  a maximum of 14 days to consider the identified CP to be in proximity.

### Actigraphy variables

Daily circadian RAR, PA, and sleep variables were calculated from the collected actigraphy data. Circadian RAR variables were computed using the *ACTman* software package^27^ by applying the NPCRA (non-parametric circadian rhythm analysis) method^28^. The NPCRA includes calculating IS (interdaily stability), IV (intradaily variability), RA (relative amplitude), M10 (average activity during the ten most active hours), and L5 (average activity during the five least active hours). All variables except IS were calculated per day, while IS required multiple days of observations and was calculated on two consecutive days of data. Additionally, we calculated cosinor RAR variables per day by applying a cosinor analysis^29,30^ to the 1-min epoch actigraphy data of each day separately. From the cosinor analysis, daily MESOR (the rhythm-adjusted mean), amplitude (difference between the rhythm’s peak and mean level), and acrophase (time of the rhythm’s peak) were estimated. MESOR can assess the daily level of physical activity, and amplitude and acrophase provide the RAR assessment. The native MotionWare software version 1.3.17 was used to calculate the sleep variables per day: time in bed (difference between going to bed and getting out of bed), sleep efficiency (ratio of total sleep time to time in bed), and fragmentation index (amount of movement during sleep). While resampling can be a valuable technique in certain contexts^31^, we chose not to employ it in this study when calculating RAR parameters to ensure that our estimations were based on the actual observed data.

### Other events

Additional information from evaluation interview logs was used to explain additional CPs not found in proximity to a depressive symptom transition. Specifically, we used data on the timing of restarted psychotropic medication after the transition and major life events measured per month. The list of threatening experiences was used to assess negative life events^32^. The list included 12 negative life event categories, for example, death or serious illness of a child, sibling, partner, close friend or relative, divorce, loss of a job or serious financial difficulties. The following list included positive life events: recovery from a serious illness of a child, sibling, partner, close friend or relative, new relationships, starting a job or financial gain, vacation. Participants indicated if a life event was present (yes) or absent (no). The last question included an open-answering option where participants could add other important life events that were not included in the questionnaire. Moreover, we used information on the timing of accelerometer replacement (i.e., due to short battery life). If an additional CP was found within 14 days of restarting psychotropic medication, a major life event, or 3 days of an accelerometer replacement, it was attributed to that particular event. It was considered undefined if a CP could not be attributed to an above-mentioned event.

### Statistical analysis

Before the analysis, all data were checked for outliers. Additionally, correlations of all variables were checked, and M10 was excluded due to its collinearity (*r* > 0.8) with MESOR.

We used the Kernel Change Point detection on the running statistics (KCP-RS) method to analyze the data^33^. KCP-RS is a non-parametric method developed to detect abrupt changes in multivariate time series. The method can detect changes in any user-specified statistic, by applying KCP on running statistics rather than raw data. Specifically, we used KCP-RS to detect change points (CPs) and their location by evaluating how similar/dissimilar the running means of the computed daily RAR, PA, and sleep variables are to each other^34^. The analysis consisted of three steps. First, the running means of each subset of predictors were obtained by sliding a time window of 7 and 14 days with a 1-day step across the time series and computing running means in each window. Second, for one up to 10 change points, the method finds optimal locations for the requested number of CPs. Third, running the KCP-RS significance test on the obtained results allows for deciding if at least one significant CP is present. If so, the optimal number of CPs was determined using a grid search. This statistical analysis was performed in R using the kcpRS package^35^. More information on the method can be found elsewhere^36^. The KCP-RS is based on daily estimates of circadian rhythm parameters; these daily values are then averaged across 7- or 14-day values during the analysis. We intentionally avoid using multiple-day windows when estimating the circadian rhythm parameters to prevent unnecessary blurring of the estimations. Applying 7- or 14-day windows as part of the KCP analysis on top of weekly estimates of circadian rhythm parameters could indeed lead to over-smoothing, which might hinder our ability to detect meaningful change points in the data.

The analysis was performed multiple times on different sets of variables (NPCRA, cosinor, sleep, and all variables) and with two sliding window sizes (7 and 14 days) for each person separately. We restricted the size of the sliding window to a multiple of seven to preserve a weekly rhythm. First, we set up a formula to combine the results from separate sub-analyses. Then, per participant, we obtained the mean location of the CPs detected by the different sub-analyses (see a detailed description in Supplementary Material 3). Next, we utilized a 10-day decision rule (CPs should be within 10 days of each other) to establish a CP by combining the identified CPs from different sub-analyses (i.e., NPCRA, cosinor, and sleep analysis); sleep variables behaved slightly differently from all other variables, and CPs identified in sleep analysis were usually more distant from CPs identified in NPCRA and cosinor analysis.

To link identified CPs to transitions, two researchers (OM and ES) compared the location of the CPs detected by KCP-RS with the transitions in depressive symptoms documented for this sample. It was also determined whether the identified CPs occurred in proximity (within 14 days before or after) to the transition in depressive symptoms^20^. The identified CPs were also compared to the location of the other events. To estimate the direction of changes, linear regression analyses were performed comparing the period before and after a CP. In the case of multiple CPs, the regression analysis was performed for the two phases around the change point.

During the analysis, we discovered that both the duration of the recording and the timing of the transition in depressive symptoms influenced how well KCP-RS can identify CPs. It requires at least several sliding windows multiple of 7 days (we selected 21 days) before and after the transition to have enough data for robust CP estimates around the time of transition. Therefore, we labeled participants with a transition in depressive symptoms differently depending on the transition's timing: “transition” and “early/late transition” participants. In these “early/late” participants, the transition in depressive symptoms happened too early (during the first 21 days, n = 3) or too late (last 21 days, n = 4) for the KCP-RS analysis to be able to identify these transitions with high power. The results for these participants are described but should be treated with caution.

## Results

The mean duration of actigraphy monitoring for included participants with sufficient data was 123 days (114—132 days). Characteristics of participants with and without a transition in depressive symptoms are given in Table 1.

**Table 1:** TRANS-ID tapering sample characteristics.

	Transition group N = 20	No transition group N = 14	*p* value
Sex (female, %)	16 (80.00)	10 (71.43)	0.564
Age, years (median, IQR)	53 (25.25)	48.5 (11.75)	0.329
Employment (yes, %)	15 (75.00)	13 (92.86)	0.311
Education, (high, %)	17 (85.00)	10 (71.43)	0.065
Relationship (yes, %)	17 (85.00)	11 (78.56)	0.631
SCL-90 across all weeks (median, IQR)	26 (14)	20 (9)	0.003
SCL-90 week 1 (median, IQR)	23.5 (9.75)	22 (10.75)	0.834

*SCL-90* symptom checklist, *IQR* interquartile range.

### Demonstration case: individual analysis

**Figure 3:**
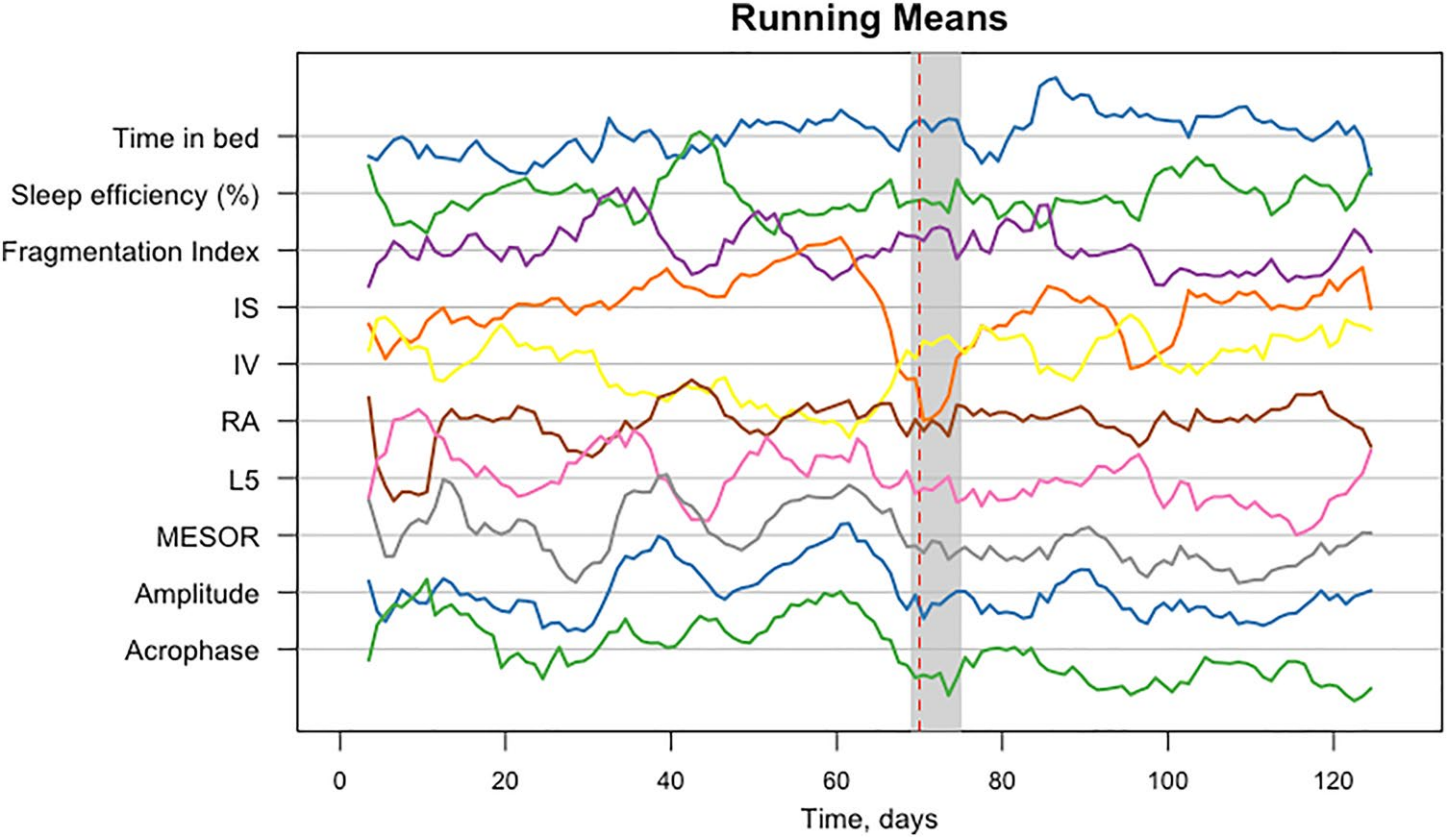
Running means for all variables, the transition period, and the Change Point (CP) for participant 1. *Note. IS* interdaily stability, *IV* intradaily variability, *RA* relative amplitude, *L5* least active 5 h, *MESOR* a rhythm-adjusted mean, *amplitude* the difference between a peak and a mean level of the rhythm, *acrophase* a moment during the day when the peak of the rhythm occurs, *time in bed* time duration between going to bed and getting out of bed, *sleep efficiency* the ratio of total sleep time to time in bed, *fragmentation index* the amount of movement or restlessness in a sleep period. Grey shaded block represents the week of transition in depressive symptoms; the red dotted line represents the identified CP.

Figure 3 shows an example of actigraphy-derived variables with an accurately identified CP near the transition in depressive symptoms. This participant had a transition in depressive symptoms around days 69–75. The CP was identified by KCP-RS around day 70. Additionally, there was a change of the actigraphy device (day 73), a major positive life event (between days 43 and 73), and restarting of antidepressant therapy (between days 75 and 104) around the same time. This means that the participant’s CP is grouped under the “Transition and LE and AR and Medication” category in Table 2 (LE—the proximity of a life event; AR—the proximity of an accelerometer replacement). Such co-occurrence happened only to this participant. The most indicative variables for this participant were: decreased IS, MESOR, amplitude, acrophase, and increased IV and time in bed. All mentioned variables, except for acrophase, changed in the expected direction based on previous group-level findings. Plots for all participants are given in Supplementary Material 4.

**Table 2:** Attribution of identified change points (CP) to transitions in depressive symptoms, major life events, replacement of actigraphy device, and restarting the medication.

Categories	Transition (n = 13)		“Early/late" transition (n = 7)		No transition (n = 14)	
	CP	%	CP	%	CP	%
Transition and other	9*	69.2	1	14.3		
Transition only	7	53.8	1	14.3		
Transition, LE and AR	1	7.7	0	0.00		
Transition, LE, AR and Medication	1	7.7	0	0.00		
Other	6	46.2	4	57.1	8	57.1
LE	5	38.5	3	42.8	3	21.4
LE and AR	0	0.00	0	0.00	1	7.1
AR	1	7.7	1	14.3	4	28.6
Undefined	3	23.1	3	42.8	9	64.3
Total	18		8		17	

*Note.* If we used a 7-day rule for combining CPs from different sub-analyses into one mean CP, we would have lost one identified CP in close proximity to a symptom transition.

*CP* number of change points, *transition* the close proximity of transition in depressive symptoms to the identified CP, *LE* the proximity of a life event, *AR* the proximity of an accelerometer replacement (see methods for details), *medication* tapering or reinitiating of psychotropic medication. Categories “Transition & LE”, “Transition and AR”, and “Transition and Medication” were not included in the table since no cases satisfied these categories. %- the percentage of identified CPs from a number of individuals in the group.

*One person had 2 CPs that could be attributed to a transition, so there were 10 CPs in total. We decided to keep 9 CPs in the table to simplify the calculation of percentages in relation to the number of individuals with and without a transition to avoid incorrect inflation of the percentages.

### Overview of identified change points

To answer the first research question, we provide an overview of all identified CPs in Table 2. In two cases (16%), identified CPs were close to multiple events simultaneously (i.e., transition in depressive symptoms, major life event, replacement of accelerometer, and re-initiation of psychotropic medication around the same time). For 69% of individuals with a transition (9/13), transitions were accompanied by a detected CP. However, one person had 2 CPs around the transition time, making 10 CPs total.

### Timing of change points

To provide approximate timing of CPs in relation to the transition timing, of the 10 identified CPs from transition participants, 7 CPs were detected after an increase in depressive symptoms, and 3 CPs were detected preceding an increase in depressive symptoms. From those preceding the onset of a transition, one CP was identified 4 days prior, one 8 days prior, and one 10 days prior to the onset of a depressive symptom transition. From those following the onset, CPs were identified within the onset week (n = 3), 1 day after the onset (n = 1), 2 days after the onset (n = 2), and 12 days after the onset (n = 1) of a depressive symptom transition. One CP in the ‘early/late’ transition participant was identified 5 days after the onset.

It is important to mention that the most responsive variables were those from the cosinor analysis (mostly MESOR and amplitude) for most participants. Additionally, KCP-RS applied to the cosinor variables resulted in equally many estimates of CPs close to transitions as KCP-RS applied to the NPCRA variables and all variables (n = 9). However, different sets of variables were more predictive for different individuals. Sleep analysis alone resulted in six estimates of CPs around depressive symptom transitions.

### Frequency of change points in individuals with and without transitions

To answer the second research question, we combined undefined and transition-related CPs (also when transition-related CPs coincide with other events) and compared the number of these CPs for individuals with and without a transition in depressive symptoms. This was done to assess whether overall changes in RAR, sleep, and physical activity occurred less frequently if participants did not develop new depressive symptoms. Results are given in Table 2. Since individuals without a transition did not have any transition-related CPs by definition, we only included undefined CPs for these individuals. We excluded CPs attributed to life events and accelerometer replacement as these CPs could be explained for each participant, and their frequencies can vary independently due to external forces. We observed 9 CPs in 14 individuals without a transition (an average of 0.64 CPs per individual) compared to 17 CPs in 20 individuals with a transition (an average of 0.85 CPs per individual, including individuals with “early/late” transitions). We used Fisher’s exact test to determine the association between the number of CPs and a transition status, and we found no statistically significant association between the two variables (*p* = 0.466). Note, that for individuals working in shifts (n = 2), there were no undefined CPs identified. When we excluded individuals with “early/late” transitions, we found 13 CPs in 13 individuals (an average of 1 CP per individual with a transition). In sum, there were relatively more CPs detected in the individuals with a transition compared to those without. However, the statistical significance of this difference could not be tested due to the limited between-person sample size.

### Direction of change in circadian rhythm variables

Results for the third research question on the direction of change in identified CPs attributed to a transition in all individuals with transitions, including those with early/late transitions, are given in Table 3. For some variables (IS, IV, MESOR, amplitude, and time in bed), we see a specific direction for most CPs associated with a transition, while others show more mixed patterns. The highest percentages were observed for a decrease in MESOR (81.81%) and amplitude (72.72%) and an increase in IV (72.72%) and time in bed (72.72%) before the transition in depressive symptoms. It is also important to point out that there was never a complete consistency in the direction of change for any variable and no clear patterns in the combination of the direction and timing of CPs. A detailed overview of the direction of changes per CP with an additional indication of the timing of each CP is given in Supplementary Material 3 Table S1. Additionally, we provided a network plot to visualize the strengths of correlations between the RAR, physical activity, and sleep variables in Supplementary Fig. S1.

**Table 3:** Direction of identified Change Points attributed to a transition (CP = 11) for all individuals with a transition in depressive symptoms.

	NPCRA								Cosinor						Sleep					
	IS		IV		RA		L5		MESOR		Amplitude		Acrophase		Time in bed		Sleep efficiency		Fragmentation index	
Expected direction*	Decrease		Increase		Decrease		Increase		Decrease		Decrease		Increase		Increase or decrease		Decrease		Increase	
Direction	−	+	−	+	−	+	−	+	−	+	−	+	−	+	−	+	−	+	−	+
All transitions (CP = 11), n																				
Total	7	4	3	8	5	6	6	5	9	2	8	3	5	6	3	8	4	7	7	4
Significant	7	3	1	4	4	3	6	2	8	1	7	1	3	4	2	8	3	3	2	3
Non-significant**	0	1	2	4	1	3	0	3	1	1	1	2	2	2	1	0	1	4	5	1

“−” refers to a decrease in the variable after a CP, “+” refers to an increase in the variable after a CP.

*IS* interdaily stability, *IV* intradaily variability, *RA* relative amplitude, *L5* least active 5 h, *MESOR* a rhythm-adjusted mean, *amplitude* the difference between a peak and a mean level of the rhythm, *acrophase* a moment during the day when the peak of the rhythm occurs, *time in bed* time duration between going to bed and getting out of bed, *sleep efficiency* the ratio of total sleep time to time in bed, *fragmentation index* the amount of movement or restlessness in a sleep period.

*Expected directions are based on results from group-level studies.

**The table displays all significant CPs related to symptom transition; therefore, non-significant directions resulted from linear regression analysis are still included.

## Discussion

In the current study, we performed repeated single-subject analyses using high-resolution actigraphy data of individuals who tapered their antidepressant medication. We assessed (1) the proximity of changes in circadian rhythm elements (RAR, PA, and sleep) to the transition in depressive symptoms, (2) the frequency of these changes in individuals with and without transitions, and (3) the direction of changes in circadian rhythm elements after the transition in depressive symptoms.

Regarding the first aim, we identified CPs at the individual level for most participants with transitions (69%, 9 out of 13). This means that most participants with transitions exhibited changes in their RAR, PA, and sleep around the transition time large enough to be detected by the change point analysis. However, the identification decreased to 50% when we included individuals with early or late transitions in the first or last 21 days of recording. Nonetheless, results from these “early/late” participants should be treated with caution since the drop in identification of CPs associated with a transition was related to the timing of transitions, making it problematic for the analysis to capture them.

Due to the novelty of the finding, no previous studies on a similar subject are available for comparison. Findings from longitudinal group-level studies indicate associations between the onset of depression and RAR, PA, and sleep^10,37^. In the current study, we observed changes in circadian rhythm elements in proximity to a depressive symptom transition in most individuals with transitions, supporting the idea that these elements are a part of the pathophysiology of depression in many individuals. Interestingly, the timing of identified changes was not consistent across individuals. In some individuals, the timing of the changes in circadian rhythm elements did not coincide with the transition in depressive symptoms, either preceding or following the transition, suggesting the link between circadian rhythm and depression may differ from person to person and even between episodes within one patient. Different sub-analyses, such as NPCRA, cosinor, and sleep analysis, showed that different sets of variables are responsive to transitions in depressive symptoms for different individuals. To summarize, the fact that we observed CPs close to a transition in most individuals with a transition suggests that RAR, PA, and sleep variables could be involved in the onset of depression. However, since these changes were not always driven by the same variables and were detected both before and after a transition, they likely contribute not in every individual and possibly in different ways, implying considerable between-individual heterogeneity.

For the second aim, we detected fewer CPs in individuals without a transition compared to those with a transition in depressive symptoms. Though the inclusion of participants with early or late transition times decreased the proportion of identified CPs, it remained higher than in participants without a transition. As hypothesized, the larger number of CPs found in participants with transitions seems to be the result of additional CPs occurring close to the transition in depressive symptoms. These additional CPs made up most of the identified CPs in the transition participants and were common enough to compensate for the fact that undefined CPs (i.e., CPs we cannot explain with available information) seemed to be somewhat less common in individuals with a transition. However, replication of these findings in a larger sample is needed to provide reliable statistical support for this finding.

For the third aim, the direction of changes was found to vary between individuals. However, some variables showed a more consistent pattern in direction than others: time in bed and IV tended to increase when depressive symptoms increased; MESOR and amplitude tended to decrease when depressive symptoms increased. However, it was difficult to estimate the direction of change in circadian rhythm variables for some individuals due to peak-like fluctuations around the transition time without a subsequent decrease or increase in variables. This could result from a decrease in depressive symptoms following shortly after an increase in symptoms (e.g., because the antidepressant medication was started shortly after symptoms returned). Nevertheless, it is an interesting observation that circadian rhythm disturbances can be so short-lived, suggesting high reactivity of circadian rhythm variables in some individuals. Therefore, it is especially crucial to examine individuals separately rather than rely on group estimates since the group estimates may be a poor reflection of what is true for most individuals in the group^13^.

Our findings align with findings from earlier group-level studies in which the majority of individuals showed a change in the direction of the examined parameters that is comparable to the most consistently found direction in group-level studies. For example, acrophase and the combination of early acrophase with a dampened 24-h activity rhythm amplitude are associated with a faster increase in depressive symptoms over time in older individuals^10^. Additionally, on average, depressed individuals exhibit lower MESOR/daytime activity, dampened amplitude, and often a delayed RAR compared to non-depressed individuals^2,3,38,39^. In the study on bipolar depression, researchers found that depressive episode days are distinguished from inter-episode states by significantly lower overall physical activity levels, a later daily onset of activity, a slightly elevated daily rhythm peak, and less evening activity^40^. Regarding the stability of the rhythm, individuals with more depressive symptoms ﻿display lower stability and higher fragmentation of the rest-activity rhythm^4,5^. Other researchers also found increased morning variability in individuals with bipolar depression compared to mixed states or mania^41^. In line with our results, in depressed individuals, reduced activity levels were accompanied by increased variability^42^. Our findings partly deviate from the group-level evidence regarding sleep duration, as depressed individuals often report either shorter or excessively longer sleep duration^7,8,43^, while we only found increased sleep duration after a transition in depressive symptoms. However, there are no examples from individual-level studies to our knowledge to compare with our findings.

This study has several strengths. This is the first study establishing actigraphy monitoring of a large number of participants for up to 4 months while they were tapering their antidepressant medication and, therefore, were at high risk for developing new depressive symptoms. As a result, a number of those individuals did develop depressive symptoms, and we could closely examine their circadian RAR, RA, and sleep for a prolonged time. This allowed us to precisely monitor the onset of clinically relevant depressive symptoms and circadian rhythm dynamics around the transition time in those individuals. Previously, researchers attempted prolonged single-subject studies. However, those included only a few participants^44^, had substantially shorter monitoring^45^, or followed only stable participants^46^. Another important and innovative aspect of the study is using the KCP-RS method. This method made it possible to statistically test for change points in the time-series data with relatively high power.

Despite the above-mentioned strengths, this study has several limitations as well. First, as the battery life of the MW8 accelerometer was limited, the device had to be replaced during data collection. This issue reduced the amount of data available for obtaining robust results from the KCP-RS analyses. Even with occurred data loss, this dataset is one of the largest and most prolonged. Second, the KCP-RS requires transitions in depressive symptoms to occur not too close (preferably 21 days) to the beginning or end of the recording for the successful identification of CPs. To tackle this issue, we categorized individuals with too early/late transitions and reported them separately, as their results should be treated with caution. Another limitation of change-point techniques is that they assume changes are sudden. If the change is gradual, change-point techniques can still detect a change, but the timing of this change reflects only one point in the process rather than the whole process of change itself. In addition, the approach of identifying transitions and change points has further limitations compared to analyzing continuous data, such as the necessity to set a threshold for CPs and transitions. This, in turn, limits the number of eligible participants for the analyses, which leads to the necessity to apply a set of thresholds and criteria specifically developed for the TRANS-ID study due to the inability to use a statistical comparison to evaluate transition/change point overlap. Third, reported re-initiation of psychotropic medication and major life events were only recorded at the month and not at the day level, making the attribution of identified CPs less reliable to certain events. Additionally, the questionnaire used to measure life events does not capture all potentially relevant life events, and the assessment was retrospective. Nevertheless, these data yielded valuable information on the impact of life events on circadian rhythm dynamics. Fourth, though all participants were in remission at baseline, some participants in this study were not completely symptom-free at baseline. This could have resulted in failure to identify CPs in such individuals with a transition in depressive symptoms who had elevated depressive symptoms at baseline if we assume that their circadian rhythms were already disrupted at baseline. Finally, the timing of detected CPs may not always correspond with a CP one would identify by visually inspecting the data. A potential cause of this is that KCP is designed to detect structural changes rather than short-lived fluctuations. This means that persistent changes that are small or only occur in a few variables can be detected using KCP, even if such changes are too subtle to be obvious during visual inspection. On the other hand, short-lived fluctuations may be visible in the graph, but these may not always indicate the kind of structural change KCP aims to detect. A larger sample would be needed to investigate circadian rhythm elements separately with sufficient power, as this may also clarify the link between detected CPs and visual inspection.

Though without concrete evidence, it is tempting to speculate about the clinical implication of the findings regarding the order of change in depressive symptoms and circadian rhythm variables. Since CPs were detected both preceding and following an increase in depressive symptoms, we can hypothesize that for different individuals, sleep and circadian rhythm can play a different role in the development of depression, with likely different mechanisms at play. Supporting this idea, in an earlier single-subject study, researchers found that ﻿the presence, nature, and direction of the temporal associations between depressive symptoms and sleep differed between individuals^46^. Our finding also clearly shows the diversity of patterns different individuals can exhibit concerning the change order. Although providing relevant information regarding circadian RAR, PA, and sleep dynamics around a depression episode, these novel results require further research to formulate solid conclusions. With the growing popularity of wrist-worn devices, it may also become possible to use data from these devices to predict and monitor real-time behavioral indicators of depression in users. Further, it is crucial to consider extra information on individuals’ life events as they can significantly impact circadian rhythm variables and explain additional circadian fluctuations. Therefore, we encourage researchers to collect more information on life events and use it to interpret the results.

To conclude, change point analysis appears promising in identifying relevant changes in circadian RAR, PA, and sleep close to depressive symptom transitions. Given the unobtrusive way of actigraphy data collection, findings from the current study show potential for detecting clinically relevant changes in depressive symptoms over an extended period. They suggest that a combination of informative and personalized circadian RAR, PA, and sleep variables could potentially provide clinically useful information. This study, however, should be seen as a proof of concept. Therefore, new methods that could prospectively detect relevant circadian rhythm changes and more empirical single-subject research would be necessary to bring this knowledge to clinical practice.

### Supplementary Information

Supplementary Information 1.

Supplementary Information 2.

Supplementary Information 3.

Supplementary Information 4.

Supplementary Information 5.

## Data Availability

The data are not publicly available due to restrictions related to data containing information that could compromise the privacy of research participants.

## References

[1] Karatsoreos IN (2014). Links between circadian rhythms and psychiatric disease. Front. Behav. Neurosci..

[2] Difrancesco S (2019). Sleep, circadian rhythm, and physical activity patterns in depressive and anxiety disorders: A 2-week ambulatory assessment study. Depress. Anxiety.

[3] Minaeva O (2020). Level and timing of physical activity during normal daily life in depressed and non-depressed individuals. Transl. Psychiatry.

[4] Luik AI, Zuurbier LA, Hofman A, Van Someren EJWW, Tiemeier H (2013). Stability and fragmentation of the activity rhythm across the sleep-wake cycle: The importance of age, lifestyle, and mental health. Chronobiol. Int..

[5] Zuurbier LA (2015). Fragmentation and stability of circadian activity rhythms predict mortality. Am. J. Epidemiol..

[6] Rebar AL (2015). A meta-meta-analysis of the effect of physical activity on depression and anxiety in non-clinical adult populations. Health Psychol. Rev..

[7] Becker NB, Jesus SN, João KADR, Viseu JN, Martins RIS (2017). Depression and sleep quality in older adults: A meta-analysis. Psychol. Health Med..

[8] Zhai L, Zhang H, Zhang D (2015). Sleep duration and depression among adults: A meta-analysis of prospective studies. Depress. Anxiety.

[9] Smagula SF (2016). Opportunities for clinical applications of rest-activity rhythms in detecting and preventing mood disorders. Curr. Opin. Psychiatry.

[10] Smagula SF (2015). Circadian rest-activity rhythms predict future increases in depressive symptoms among community-dwelling older men. Am. J. Geriatr. Psychiatry Off. J. Am. Assoc. Geriatr. Psychiatry.

[11] Strawbridge WJ, Deleger S, Roberts RE, Kaplan GA (2002). Physical activity reduces the risk of subsequent depression for older adults. Am. J. Epidmiol..

[12] Schuch FB (2018). Physical activity and incident depression: A meta-analysis of prospective cohort studies. Am. J. Psychiatry.

[13] Zuidersma M (2020). Single-subject research in psychiatry: Facts and fictions. Front. Psychiatry.

[14] Fisher AJ, Medaglia JD, Jeronimus BF (2018). Lack of group-to-individual generalizability is a threat to human subjects research. Proc. Natl. Acad. Sci. U.S.A..

[15] Smit, A. C., Helmich, M. A., Snippe, E. & Wichers, M. Transitions in depression (TRANS-ID) tapering: Study protocol for a repeated intensive longitudinal n= 1 study design to search for personalized early warning. *OSF* Preprint at (2020).

[16] van de Water ATMM, Holmes A, Hurley DA (2011). Objective measurements of sleep for non-laboratory settings as alternatives to polysomnography—A systematic review. J. Sleep Res..

[17] Hills AP, Mokhtar N, Byrne NM (2014). Assessment of physical activity and energy expenditure: An overview of objective measures. Front. Nutr..

[18] Doherty A (2018). Circadian rhythms and mental health: Wearable sensing at scale. Lancet Psychiatry.

[19] Geddes JR (2003). Relapse prevention with antidepressant drug treatment in depressive disorders: A systematic review. Lancet.

[20] Smit AC, Snippe E, Bringmann LF, Hoenders HJR, Wichers M (2022). Transitions in depression: If, how, and when depressive symptoms return during and after discontinuing antidepressants. Qual. Life Res..

[21] Kunkels YK (2023). Risk ahead: Actigraphy-based early-warning signals of increases in depressive symptoms during antidepressant discontinuation. Clin. Psychol. Sci..

[22] Derogatis, L. R. & Unger, R. Symptom checklist-90-revised. In *The Corsini Encyclopedia of Psychology* (John, 2010). 10.1002/9780470479216.corpsy0970.

[23] Gupta NJ, Khare A (2020). Disruption in daily eating-fasting and activityrest cycles in Indian adolescents attending school. PLoS ONE.

[24] Comiran Tonon A (2022). Handling missing data in rest-activity time series measured by actimetry. Chronobiol. Int..

[25] van Buuren S, Groothuis-Oudshoorn K (2011). mice: Multivariate imputation by chained equations in R. J. Stat. Softw..

[26] Di J (2022). Considerations to address missing data when deriving clinical trial endpoints from digital health technologies. Contemp. Clin. Trials.

[27] Kunkels YK (2020). ACTman: Automated preprocessing and analysis of actigraphy data. J. Sci. Med. Sport.

[28] van Someren EJW, Kessler A, Mirmiran M, Swaab DF (1997). Indirect bright light improves circadian rest-activity rhythm disturbances in demented patients. Biol. Psychiatry.

[29] Nelson W (1979). Methods for cosinor-rhythmometry. Chronobiologia.

[30] Cornélissen G (2014). Cosinor-based rhythmometry. Theor. Biol. Med. Model..

[31] Gonçalves BSB, Adamowicz T, Louzada FM, Moreno CR, Araujo JF (2015). A fresh look at the use of nonparametric analysis in actimetry. Sleep Med. Rev..

[32] Brugha T, Bebbington P, Tennant C, Hurry J (1985). The list of threatening experiences: A subset of 12 life event categories with considerable long-term contextual threat. Psychol. Med..

[33] Cabrieto J (2018). Capturing correlation changes by applying kernel change point detection on the running correlations. Inf. Sci..

[34] Cabrieto J, Tuerlinckx F, Kuppens P, Grassmann M, Ceulemans E (2017). Detecting correlation changes in multivariate time series: A comparison of four non-parametric change point detection methods. Behav. Res. Methods.

[35] Cabrieto J (2021). kcpRS: An R package for performing kernel change point detection on the running statistics of multivariate time series. Behav. Res. Methods.

[36] Cabrieto J, Adolf J, Tuerlinckx F, Kuppens P, Ceulemans E (2018). Detecting long-lived autodependency changes in a multivariate system via change point detection and regime switching models. Sci. Rep..

[37] Crouse JJ (2021). Circadian rhythm sleep–wake disturbances and depression in young people: Implications for prevention and early intervention. Lancet Psychiatry.

[38] Hori H (2016). 24-h activity rhythm and sleep in depressed outpatients. J. Psychiatr. Res..

[39] Burton C (2013). Activity monitoring in patients with depression: A systematic review. J. Affect. Disord..

[40] Gershon A, Ram N, Johnson SL, Harvey AG, Zeitzer JM (2016). Daily actigraphy profiles distinguish depressive and interepisode states in bipolar disorder. Clin. Psychol. Sci. J. Assoc. Psychol. Sci..

[41] Scott J, Vaaler AE, Fasmer OB, Morken G, Krane-Gartiser K (2017). A pilot study to determine whether combinations of objectively measured activity parameters can be used to differentiate between mixed states, mania, and bipolar depression. Int. J. Bipolar Disord..

[42] Krane-Gartiser K, Henriksen TEG, Morken G, Vaaler A, Fasmer OB (2014). Actigraphic assessment of motor activity in acutely admitted inpatients with bipolar disorder. PLoS ONE.

[43] Kaneita Y (2006). The relationship between depression and sleep disturbances: A Japanese nationwide general population survey. J. Clin. Psychiatry.

[44] Wichers M, Smit AC, Snippe E (2020). Early warning signals based on momentary affect dynamics can expose nearby transitions in depression: A confirmatory single-subject time-series study. J. Person Oriented Res..

[45] McFadden T, Fortier MS, Guérin E (2017). Investigating the effects of physical activity counselling on depressive symptoms and physical activity in female undergraduate students with depression: A multiple baseline single-subject design. Ment. Health Phys. Act..

[46] Zuidersma M (2021). Temporal dynamics of depression, cognitive performance and sleep in older persons with depressive symptoms and cognitive impairments: A series of eight single-subject studies. Int. Psychogeriatr..

[47] Cabrieto J, Tuerlinckx F, Kuppens P, Hunyadi B, Ceulemans E (2018). Testing for the presence of correlation changes in a multivariate time series: A permutation based approach. Sci. Rep..

