# Supplementary material for: Individual-specific change points in circadian rest-activity rhythm and sleep in individuals tapering their antidepressant medication: an actigraphy study
Source: Sci Rep. 2024 Jan 9;14:855. doi: 10.1038/s41598-023-50960-1 (PMC10776866; doi:10.1038/s41598-023-50960-1)
Supplement: Supplementary file 1 — Supplementary Information 1. [file 41598_2023_50960_MOESM1_ESM.docx]

**Deviations from the preregistration “Modeling individual-specific changes in circadian rest-activity rhythm before and during a depressive episode”**

1. In the preregistration of this study, we formulated two additional research questions regarding the exact timing of circadian rhythm changes (1. Do changes in RAR, PA, and sleep variables precede recurrence of depression symptoms, occur during this transition, or follow it? 2. Are there individual differences in the timing of these changes?). These questions were not feasible to answer with the analysis we utilized in the study due to using a sliding window approach that obscures identification of the exact timing of changes. Instead, we focused on identifying change points in close proximity to a transition in depressive symptoms.
2. We excluded the ESM item “I have slept well last night”, which was assessed once a day in the first ESM questionnaire of the day. This item was supposed to be a subjective sleep quality variable. Instead, we used a fragmentation index variable from the sleep analysis in the MotionWare software to assess objective sleep quality. We decided not to combine ESM and actigraphy variables into one sleep analysis because the potential misalignment of objective and subjective variables.
3. We did not compute the starting point of M10 since this variable is very similar by its concept and calculation to acrophase.
4. In the preregistration, we stated that we would exclude the data of the whole day (24 hours) if more than *three* consecutive hours missing during the day. In the paper, we changed it from *three* to *four* hours because this initial cut-off was too stringent and was causing excessive data loss. Since this was becoming a problem with a demanding analysis and the initial choice for the cut-off of three hours was based on one article with the same cut-off, we decided to modify out initial cut-off to four hours.
5. In the preregistration, we split individuals into three different groups: sudden, gradual and no transition groups. However, in the paper, we merged sudden and gradual transition groups since analysing them separately did not contribute to answering the stated research questions.
