# Supplementary material for: Individual-specific change points in circadian rest-activity rhythm and sleep in individuals tapering their antidepressant medication: an actigraphy study
Source: Sci Rep. 2024 Jan 9;14:855. doi: 10.1038/s41598-023-50960-1 (PMC10776866; doi:10.1038/s41598-023-50960-1)
Supplement: Supplementary file 2 — Supplementary Information 2. [file 41598_2023_50960_MOESM2_ESM.docx]

**Supplementary Material 2. An R-script and example plots of imputed data**

Preparations: - remove top info,

- remove the first and the last incomplete day up until 00:00,

- replace missing bits for imputation with NA

# Load the required packages

library(mice)

library(lattice)

library(dplyr)

# Load the data

setwd("…")

data <- read.csv("….csv", header=T, sep=",")

colnames(data)[3] <- "Activity"

data$Imputed <- NULL

# Create a vector of variables to be imputed, with a predictor variable time

data$Date <- as.Date(data$Date, "%d-%m-%Y")

date <- unique(data$Date)

times <- data %>% filter(Date == date[1]) %>% select(Time)

data$time <- rep(c(1:nrow(times)), length(date))

# Imputing missing data

missing_data <- data.frame(data$Activity, data$time) # time as predictor

tempdata <- mice(missing_data, m = 10, maxit = 5, method = "rf")

## Visualise

start <- "2018-03-01" #change depending on the imputed data is located

end <- "2018-03-03"

# Original data with missing

missing_data$date <- data$Date

missing_data_ <- missing_data %>% filter(between(date, as.Date(start), as.Date(end)))

raw_data_plot <- plot(missing_data_$data.Activity,

col = "darkred",

cex = 0.70,

main = "Raw dataset",

xlab = "Cumulative frequency of 30-second epochs",

ylab = "Activity counts")

abline(h = 1800, lty = "twodash", lwd = 2)

# Imputed data

completedata <- complete(tempdata, 1)

completedata$date <- data$Date

completedata_ <- completedata %>% filter(between(date, as.Date(start), as.Date(end)))

imputed_data_plot <- plot(completedata_$data.Activity,

col = "darkblue",

cex = 0.70,

main = "Imputed dataset",

xlab = "Cumulative frequency of 30-second epochs",

ylab = "Activity counts")

abline(h = 1800, lty = "twodash", lwd = 2)


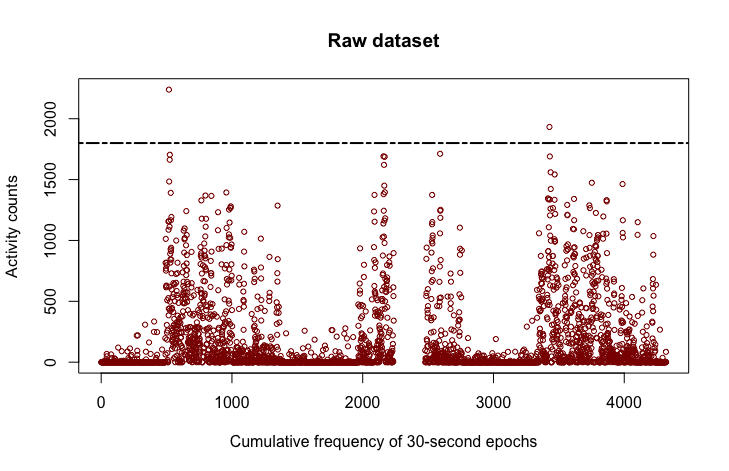


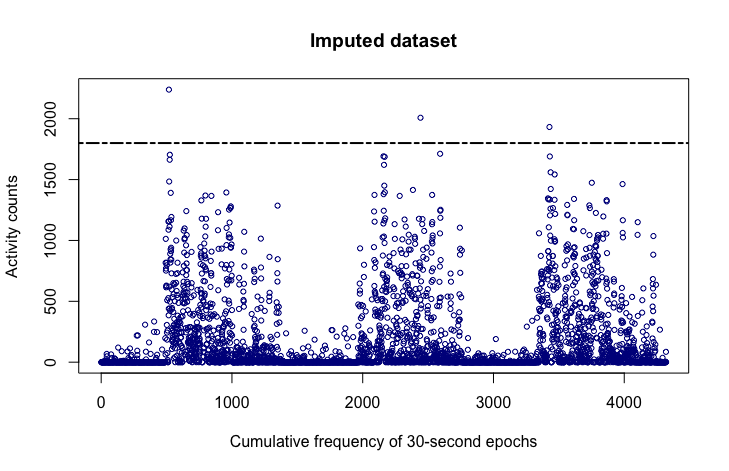


**Figure S1. Example of raw (red) and imputed actigraphy data (blue)**
