# Supplementary material for: Individual-specific change points in circadian rest-activity rhythm and sleep in individuals tapering their antidepressant medication: an actigraphy study
Source: Sci Rep. 2024 Jan 9;14:855. doi: 10.1038/s41598-023-50960-1 (PMC10776866; doi:10.1038/s41598-023-50960-1)
Supplement: Supplementary file 3 — Supplementary Information 3. [file 41598_2023_50960_MOESM3_ESM.docx]

**Supplementary Material 3. Detailed description of all identified Change Points and decision rules for all participants**

**Table S1. A detailed description of the direction and timing of transition-related CPs**

| CP# | IS | IV | RA | L5 | MESOR | Amplitude | Acrophase | Time in bed | Sleep efficiency | Fragmentation index |
| --- | --- | --- | --- | --- | --- | --- | --- | --- | --- | --- |
| 1 | -1 | 1 | -1 | 1 | -1 | -1 | 1 | 1 | -1 | 1 |
| 2 | 0 | 1 | -1 | -1 | -1 | -1 | 1 | 1 | 0 | 0 |
| 3 | 1 | 0 | 1 | -1 | 0 | 0 | 0 | 0 | 1 | -1 |
| 4 | 1 | -1 | 1 | -1 | -1 | 0 | 1 | 1 | 1 | 0 |
| 5 | -1 | 1 | 0 | -1 | -1 | -1 | -1 | 1 | 0 | -1 |
| 6 | -1 | 0 | 0 | 0 | 1 | 1 | -1 | -1 | -1 | 1 |
| 7 | -1 | 0 | -1 | 0 | -1 | -1 | 1 | -1 | 0 | 0 |
| 8 | -1 | 0 | 1 | -1 | 0 | 0 | 0 | 1 | 0 | 0 |
| 9 | -1 | 1 | 0 | -1 | -1 | -1 | -1 | 1 | 1 | 0 |
| 10 | 1 | 0 | -1 | 1 | -1 | -1 | 0 | 1 | -1 | 1 |
| 11 | -1 | 0 | 0 | 0 | -1 | -1 | 0 | 1 | 0 | 0 |

Note. CP - change point; IS – interdaily stability; IV – intradaily variability; RA – relative amplitude; L5 –least active 5 hours; MESOR – a rhythm-adjusted mean; amplitude – the difference between a peak and a mean level of the rhythm; acrophase - a moment during the day when the peak of the rhythm occurs; time in bed – time duration between going to bed and getting out of bed; sleep efficiency - the ratio of total sleep time to time in bed; fragmentation index – the amount of movement or restlessness in a sleep period; more details on the variables are given in the Method section; -1 – decrease; 1 – increase; 0 – non-significant; blue highlight - CP identified before the onset of a transition; yellow highlight - CP identified during the onset week; green highlight - CP identified after the onset of a transition.

**Decision rules for selecting CPs and their locations**

We considered a CP only if it occurred more than 2 times on separate sub-analyses. CPs should be within 10 days of each other in different sub-analyses to be connected. In case a CP occurred only 2 times, we checked significance of this CP location for other variables. If there are CPs with the same location and p-value between >0.05 and <0.1, include this CP.

If there is a CP that can be matched with more CPs (but those not with each other), match with the one of all variables

For participants with only 14-day sliding window, we considered a CP if it occurred more than 1 time; if it occurred only 1 time we checked significance of this CP location for other variables. If there are CPs with the same location and p-value between >0.05 and <0.1, include this CP.

**Table S2. A detailed description of all identified Change Points for all participants**

**Notes. ^1^ Bold** – CP close to a transition; *Italic* – CP close to a life event; Underscore – CP close to an accelerometer replacement; ~~Strikethrough~~ – undefined CP. Combination of these indications means that multiple indication apply to the identified CP. ^2^ Grey color means p-value is >0.05 but <0.1

| **No** | **Days before Transition** | **No. act days** | **CP locations** | **# CP** | **Proximity transition** | | | **NPCRA 1 day** | | **NPCRA 2 days** | | **Cosinor analysis** | | **Sleep variables** | | **All variables** | |  |
| --- | --- | --- | --- | --- | --- | --- | --- | --- | --- | --- | --- | --- | --- | --- | --- | --- | --- | --- |
|  |  |  |  |  | +/-3 days | +/-7 days | +/-14 days | **7-days window** | **14-days window** | **7-days window** | **14-days window** | **7-days window** | **14-days window** | **7-days window** | **14-days window** | **7-days window** | **14-days window** |  |
| **Transition participants** | | | | | | | | | | | | | | | | | | |
| 1 | 57-63 | 123 | - | 0 |  |  |  | no | no | no | no | no | no | no | no | no | no |  |
| 2 | 64-70 | 123 | *106^1^* | 1 |  |  |  | 106 | no | 106 | no | 104 | 95^2^ | 47, 67, 80, 117 | 47 | 109 | 87 |  |
| 3 | 44-50 | 122 | **52** | 1 | 1 | 0 | 0 | - | no | - | 50 | - | 55 | - | no | - | 76 |  |
| 4 | 74-80 | 119 | *38*, **82**, *100* | 3 | 1 | 0 | 0 | 94 | 87 | 84 | 86 | 37 | 40 | 16, 39, 56, 101 | no | 16, 37, 66, 79, 101 | 98 |  |
| 5 | 54-60 | 114 | ~~33~~, **57** | 2 | 1 | 0 | 0 | no | no | 100 | no | 32, 58 | 30 | 56 | 55 | 33, 57 | 55 |  |
| 6 | 39-45 | 122 | **31** | 1 | 0 | 0 | 1 | 29 | 29 | 31, 53 | 33 | 30, 99 | no | 35 | no | 31 | 34 |  |
| 7 | 69-75 | 124 | ***70*** | 1 | 1 | 0 | 0 | 68 | 36, 71 | 34, 68 | 70 | 68 | 72 | no | no | 69 | 72 |  |
| 8 | 73-79 | 123 | ~~48~~, *107* | 2 |  |  |  | 47 | 50 | no | no | 47, 98 | 50 | 110 | no | 47, 107 | 51, 104 |  |
| 9 | 30-36 | 118 | **48**, 60 | 2 | 0 | 0 | 1 | 46, 60 | no | no | no | 42, 48, 58 | no | 50, 60 | 63 | 46, 59 | 63 |  |
| 10 | 39-45 | 130 | **35**, ~~108~~ | 2 | 0 | 1 | 0 | 106 | 35, 110 | no | 35, 110 | 34, 108 | 34, 111 | 105 | 109 | 106 | 110 |  |
| 11 | 61-67 | 119 | **68,** *93* | 2 | 1 | 0 | 0 | 71 | 91 | 95 | 93 | 96 | 92 | 62 | 60 | 71 | 93 |  |
| 12 | 51-57 | 124 | ***41***, **56** | 2 | 1 | 0 | 1 | no | 42 | 41, 49 | 39, 53 | 57 | 58 | 64 | 42 | 53 | 58 |  |
| 13 | 40-46 | 124 | - | 0 |  |  |  | no | no | no | no | no | no | 71 | 55 | no | no |  |
| **Early/late transition participants** (transition on the first/last 21 days) | | | | | | | | | | | | | | | | | | |
| 1 | 107-113 | 123 | ~~48~~ | 1 |  |  |  | 94 | 106 | 93 | 104 | 50 | 47 | 108 | no | 69 | 47, 106 |  |
| 2 | 18-24 | 132 | ~~92~~ | 1 |  |  |  | no | no | 94 | no | no | no | 88 | 90 | 94 | 92 |  |
| 3 | 12-18 | 132 | **23**, *113* | 2 | 0 | 1 |  | 21 | 23, 110 | 21 | 23, 110 | 21, 115 | 22, 114 | 82 | 59 | 23, 114 | 25, 85, 114 |  |
| 4 | 19-25 | 119 | - | 0 |  |  |  | no | no | no | no | 21, 48 | no | no | no | 68 | no |  |
| 5 | 111-117 | 123 | *30* | 1 |  |  |  | 30 | 36, 55 | 29 | 32 | 27 | 31 | 27 | no | 29 | 31 |  |
| 6 | 11-17 | 122 | 55 | 1 |  |  |  | - | 55 | - | no | - | no | - | no | - | 55 |  |
| 7 | 108-114 | 123 | *52*, ~~64~~ | 2 |  |  |  | 64 | no | 63 | 66 | 51 | 50 | 38 | 41 | 52 | 56 |  |
| **No transition participants** | | | | | | | | | | | | | | | | | | |
| 1 | NA | 111 | 42 | 1 |  |  |  | no | 48 | 41 | 42 | 43 | 44 | 23, 37 | 39 | 44 | 42 |  |
| 2 | NA | 123 | ~~20~~ | 1 |  |  |  | 20 | no | 20, 47, 59, 103 | no | 20 | no | no | no | 21, 37, 58, 104 | no |  |
| 3 | NA | 96 | 72 | 1 |  |  |  | no | no | 75 | 76 | no | no | 70 | 71 | 78 | 45 |  |
| 4 | NA | 122 | - | 0 |  |  |  | no | no | 85 | 35 | no | no | 109 | no | no | no |  |
| 5 | NA | 116 | ~~52~~, 68 | 2 |  |  |  | 50, 68 | 48 | no | no | 49 | 49 | 20, 53, 68 | no | 51, 68 | 53, 69 |  |
| 6 | NA | 124 | *50* | 1 |  |  |  | 47 | 50 | 53 | 50 | 55 | 51 | 72 | 102 | 71 | 50, 78, 101 |  |
| 7 | NA | 118 | ~~68, 100~~ | 2 |  |  |  | 87 | 88 | 68 | 67 | 98 | 67 | 101 | no | 100 | 68 |  |
| 8 | NA | 123 | - | 0 |  |  |  | no | no | no | no | 16 | no | no | no | 17 | no |  |
| 9 | NA | 119 | ~~27~~, 47 | 2 |  |  |  | 21 | 19 | 30 | 29 | no | no | 45 | 48 | 45 | 48 |  |
| 10 | NA | 122 | *87* | 1 |  |  |  | 87 | no | no | 87 | 87 | 86 | 40 | 39 | 60, 87, 95 | 86 |  |
| 11 | NA | 123 | ~~48~~ | 1 |  |  |  | 48 | 46 | 47 | 46 | 48 | 52 | no | no | 48 | 46 |  |
| 12 | NA | 117 | ~~33, 81~~ | 2 |  |  |  | 33, 82 | 27 | 35, 80 | 39, 83 | 56, 80 | 55 | no | no | 32, 81 | no |  |
| 13 | NA | 123 | *60*, *78,* ~~97~~ | 3 |  |  |  | 96 | 93 | 62, 73, 88 | 58, 82 | 59, 76 | 83 | 61, 99 | 64, 103 | 60, 76 | 59, 79 |  |
| 14 | NA | 122 | - | 0 |  |  |  | no | 80 | 94 | no | no | no | no | no | no | 79 |  |
| **Excluded participants** (too short recording (less than 90 days) or transition before or after actigraphy recording) | | | | | | | | | | | | | | | | | | |
| 1 | NA | 43 | - | 0 |  |  |  | no | no | no | no | no | no | no | no | no | no |  |
| 2 | NA | 41 | - | 0 |  |  |  | no | 20 | no | no | 20 | 21 | no | 21 | no | 20 |  |
| 3 | NA | 51 | - | 0 |  |  |  | no | no | 9 | no | no | no | no | no | no | no |  |
| 4 | NA | 54 | - | 0 |  |  |  | no | no | 24 | 23 | no | no | no | no | no | no |  |
| 5 | NA | 72 | - | 0 |  |  |  | - | no | - | no | - | no | - | no | - | no |  |
| 6 | 4-10 | 76 | - | 0 |  |  |  | no | no | 37 | 36 | no | no | no | no | no | no |  |
| 7 | 12-18 | 33 | - | 0 |  |  |  | 16 | no | 15 | no | 16 | no | no | no | 16 | no |  |
| 8 | 64-70 | 67 | ~~35~~ | 1 |  |  |  | no | no | 38 | no | 36 | 34 | no | 24 | no | 34 |  |
| 9 | transition after act end | 89 | 37, *58* | 1 |  |  |  | no | no | 38 | no | no | no | 59 | 57 | 38, 59 | 36, 58 |  |
| 10 | transition before act start | 46 | - | 0 |  |  |  | 18 | no | 15 | no | no | no | no | no | no | no |  |
| 11 | transition after act end | 104 | *51* | 1 |  |  |  | no | no | no | no | 50 | 54 | no | no | 50 | no |  |
| 12 | transition after act end | 123 | ~~63~~, ~~80~~ | 2 |  |  |  | 63 | 61 | 63 | 66 | 81 | 78 | 73 | no | 84 | 78 |  |
| 13 | transition after act end | 106 | ~~45~~ | 1 |  |  |  | no | no | no | no | 50 | no | 42 | 45 | 50, 76 | 47, 77 |  |
| 14 | transition after act end | 120 | - | 0 |  |  |  | no | no | no | no | no | no | no | no | no | no |  |
| 15 | transition after act end | 118 | ~~42~~, ~~82~~ | 2 |  |  |  | 39, 83 | 42, 87 | no | no | 83 | 82 | 75 | no | 85 | 44, 80 |  |
| 16 | transition after act end | 121 | ~~34~~, *98* | 2 |  |  |  | 33, 97 | 37, 97 | 33, 98 | 36, 97 | 33, 100 | 50, 99 | 100 | 99 | 33, 97 | 35, 97 |  |
