# Supplementary material for: Individual-specific change points in circadian rest-activity rhythm and sleep in individuals tapering their antidepressant medication: an actigraphy study
Source: Sci Rep. 2024 Jan 9;14:855. doi: 10.1038/s41598-023-50960-1 (PMC10776866; doi:10.1038/s41598-023-50960-1)
Supplement: Supplementary file 4 — Supplementary Information 4. [file 41598_2023_50960_MOESM4_ESM.docx]

**Supplementary Material 4. Plots of all participants**

**Notes. ^1^ Bold** – CP close to a transition; *Italic* – CP close to a life event; Underscore – CP close to an accelerometer replacement; ~~Strikethrough~~ – undefined CP. Combination of these indications means that multiple indication apply to the identified CP. Grey shaded block represents the week of transition in depressive symptoms; red dotted line represents identified CPs.

Abbreviations: IS – interdaily stability; IV – intradaily variability; RA – relative amplitude; L5 –least active 5 hours; MESOR – a rhythm-adjusted mean; amplitude - difference between a peak and a mean level of the rhythm; acrophase - a moment during the day when the peak of the rhythm occurs; time in bed – time duration between going to bed and getting out of bed; sleep efficiency - the ratio of total sleep time to time in bed; fragmentation index - amount of movement or restlessness in a sleep period.

| **No** | **Days before Transition** | **No actigraphy days** | **CP locations** | **Plot** |
| --- | --- | --- | --- | --- |
| **Transition participants** | | | | |
| 1 | 57-63 | 123 | - | 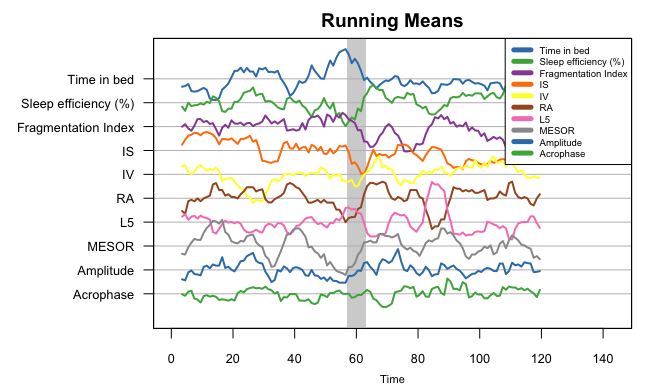 |
| 2 | 64-70 | 123 | *106*^1^ | 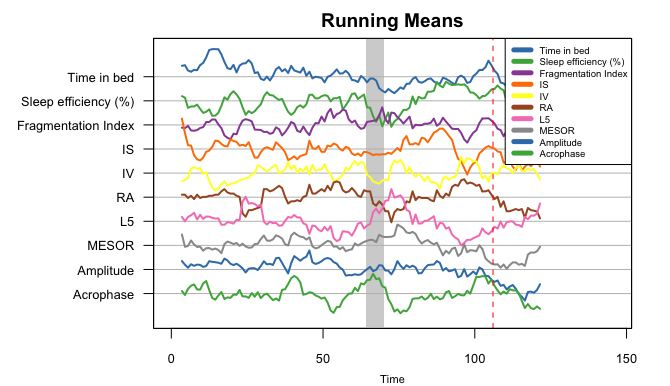 |
| 3 | 44-50 | 122 | 52 | 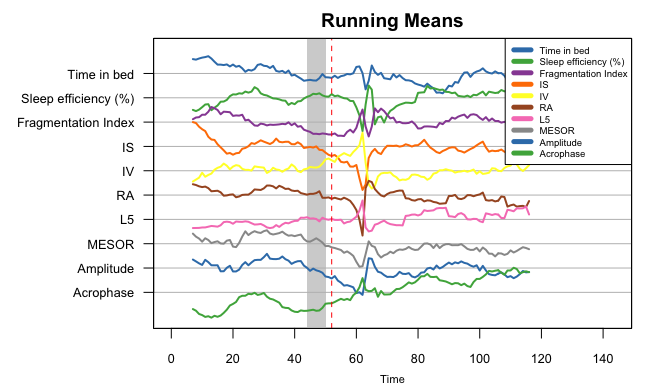 |
| 4 | 74-80 | 119 | *38*, **82**, *100* | 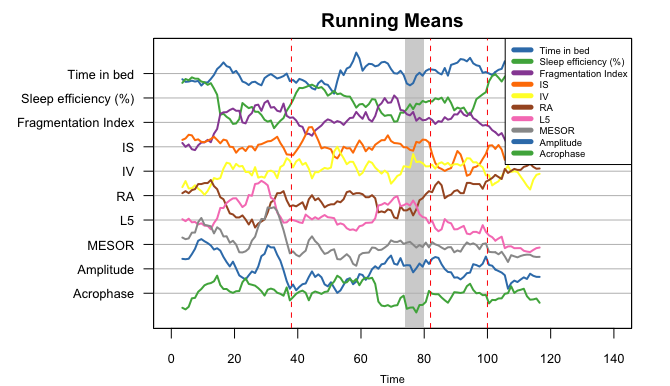 |
| 5 | 54-60 | 114 | ~~33~~, **57** | 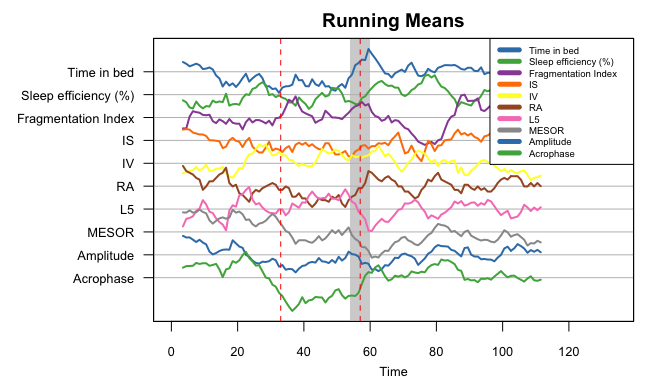 |
| 6 | 39-45 | 122 | **31** | 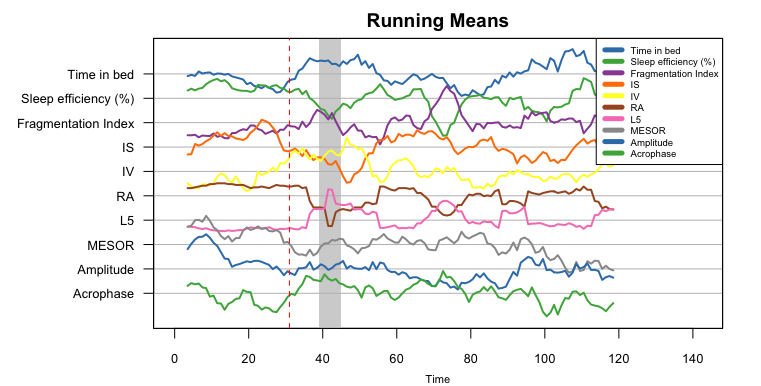 |
| 7 | 69-75 | 124 | ***70*** | 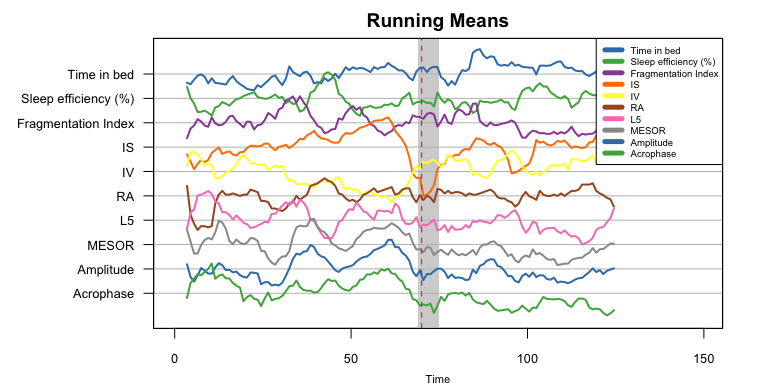 |
| 8 | 73-79 | 123 | ~~48~~, *107* | 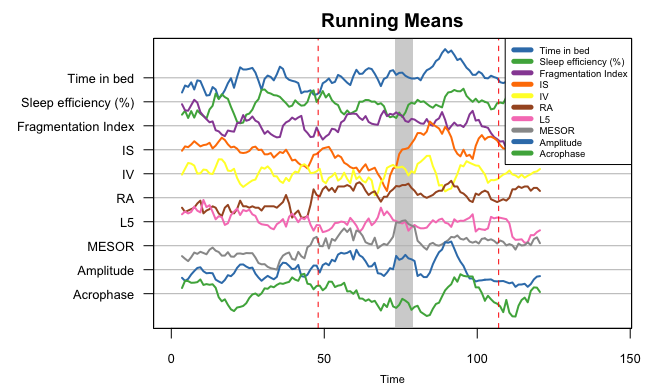 |
| 9 | 30-36 | 118 | **48**, 60 | 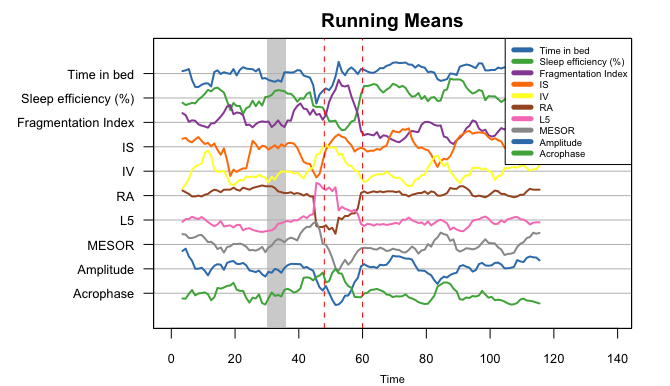 |
| 10 | 39-45 | 130 | **35**, ~~108~~ | 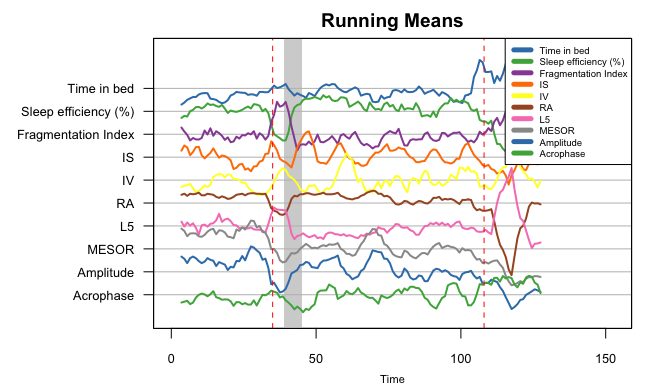 |
| 11 | 61-67 | 119 | **68,** *93* | 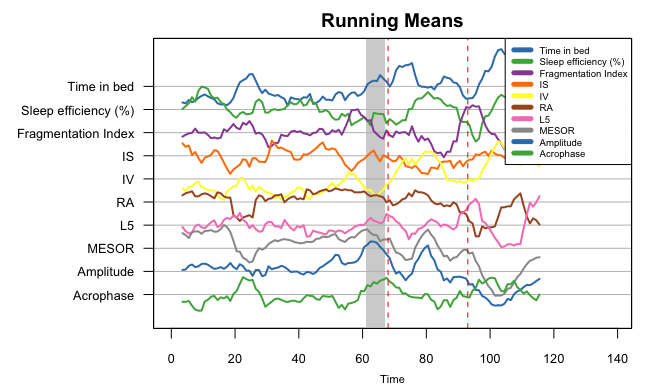 |
| 12 | 51-57 | 124 | ***41***, **56** | 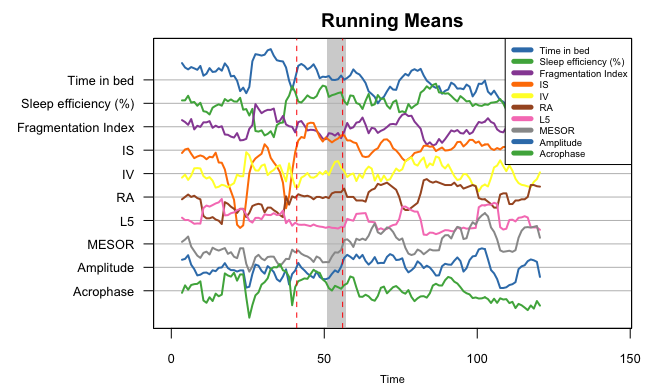 |
| 13 | 40-46 | 124 | - | 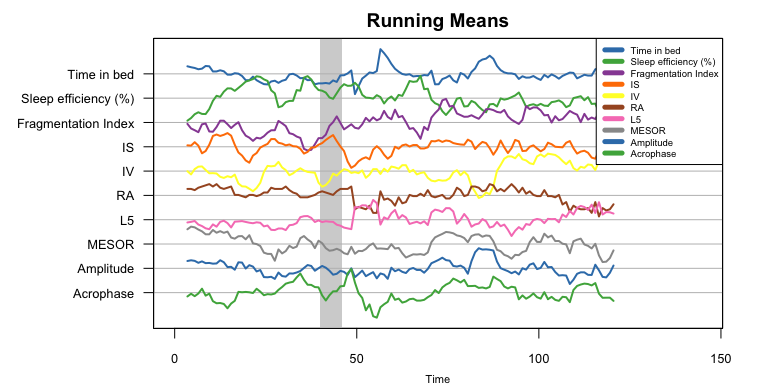 |
| **Early/late transition participants** (transition on the first/last 21 days) | | | | |
| 1 | 107-113 | 123 | ~~48~~ | 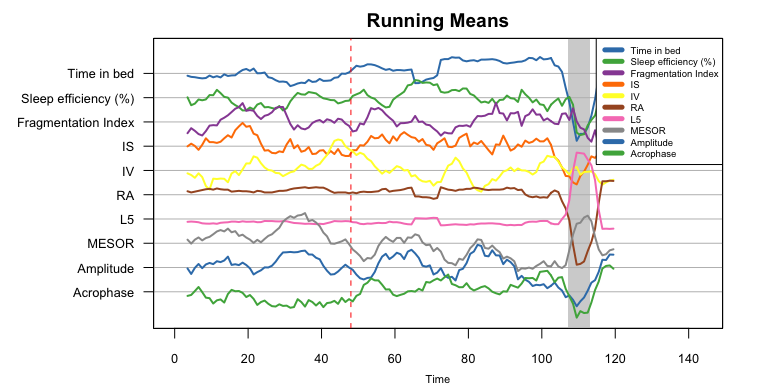 |
| 2 | 18-24 | 132 | ~~92~~ | 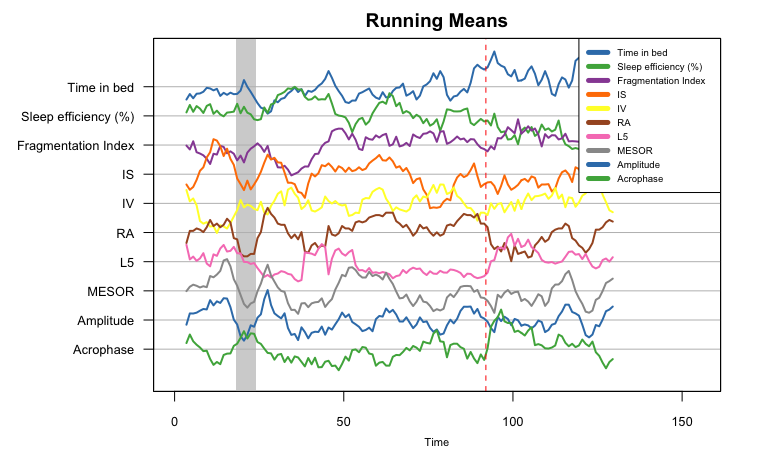 |
| 3 | 12-18 | 132 | **23**, *113* | 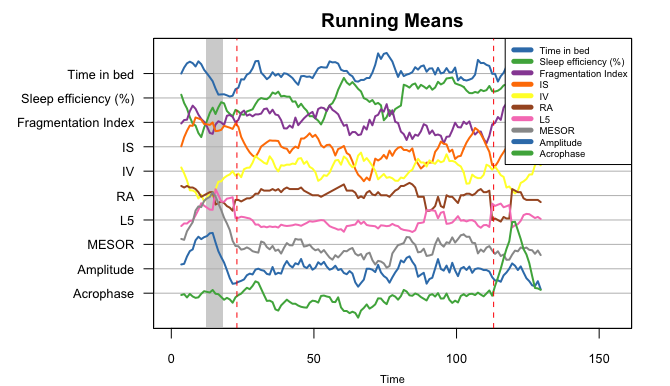 |
| 4 | 19-25 | 119 | - | 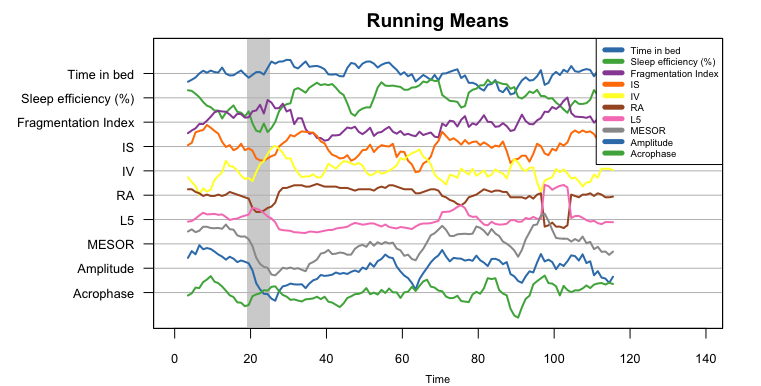 |
| 5 | 111-117 | 123 | *30* | 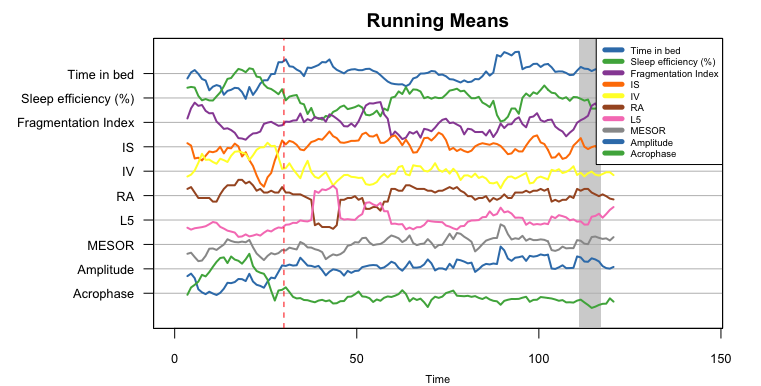 |
| 6 | 11-17 | 122 | 55 | 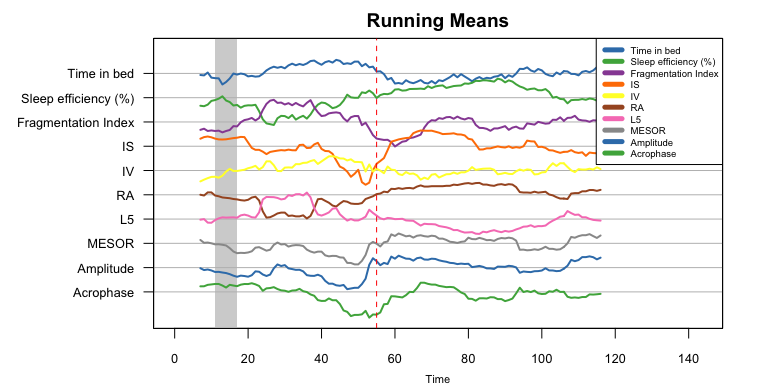 |
| 7 | 108-114 | 123 | *52*, ~~64~~ | 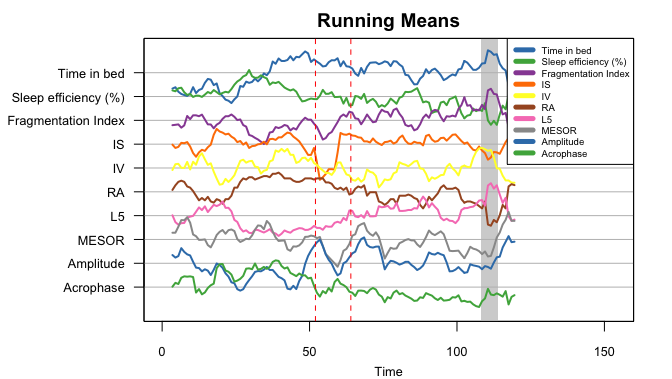 |
| **No transition participants** | | | | |
| 1 | NA | 111 | 42 | 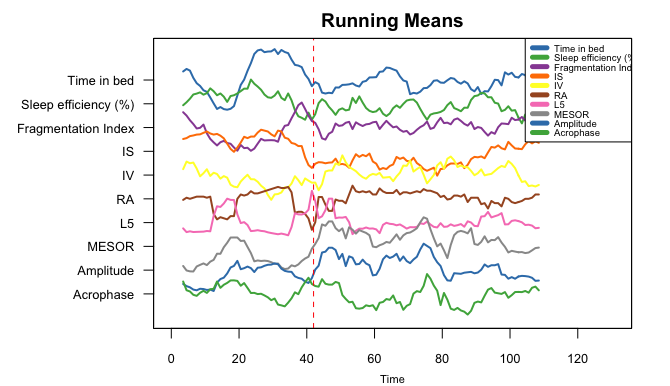 |
| 2 | NA | 123 | ~~20~~ | 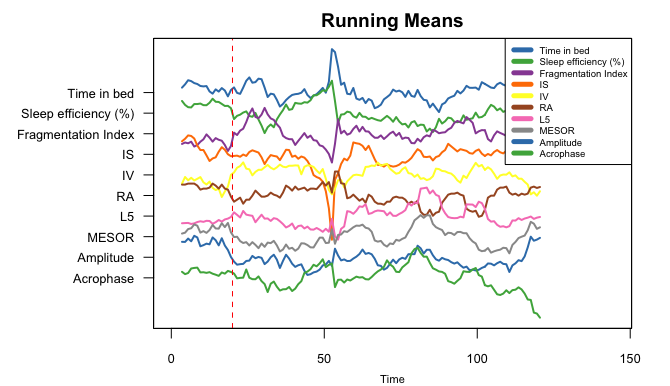 |
| 3 | NA | 96 | 72 | 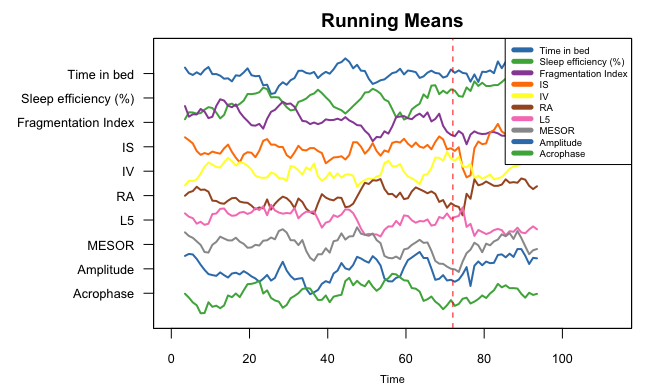 |
| 4 | NA | 122 | - | 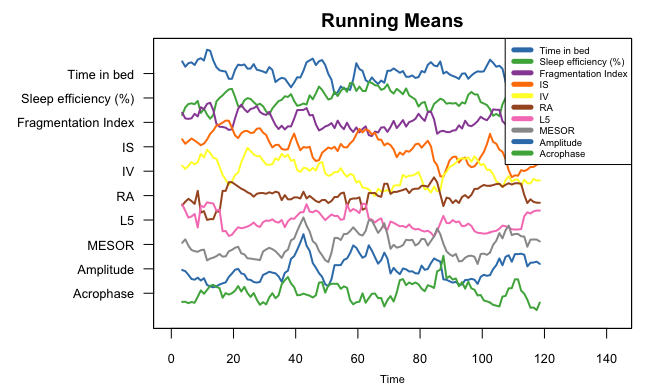 |
| 5 | NA | 116 | ~~52~~, 68 | 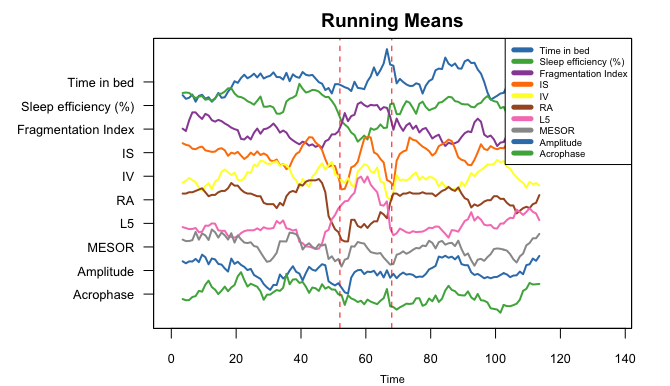 |
| 6 | NA | 124 | *50* | 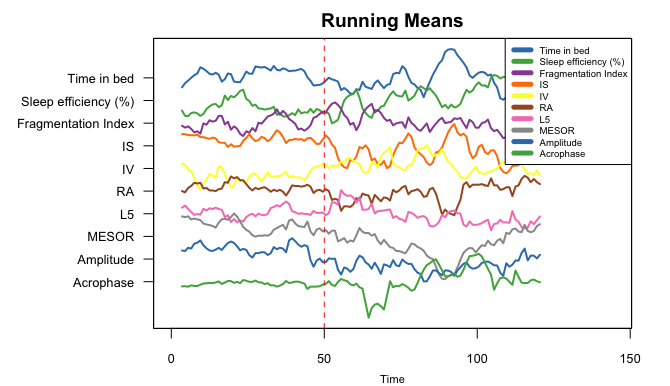 |
| 7 | NA | 118 | ~~68, 100~~ | 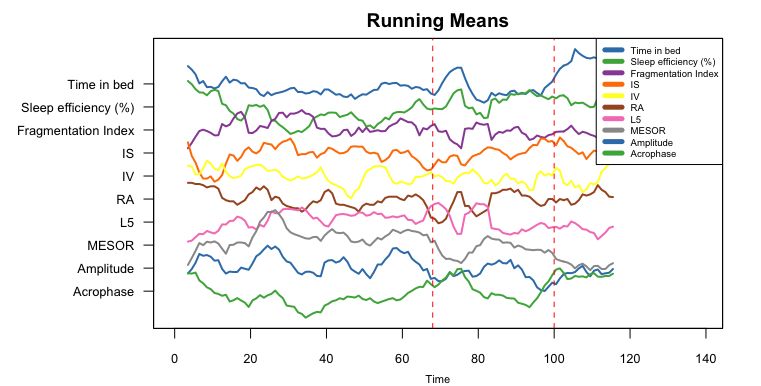 |
| 8 | NA | 123 | - | 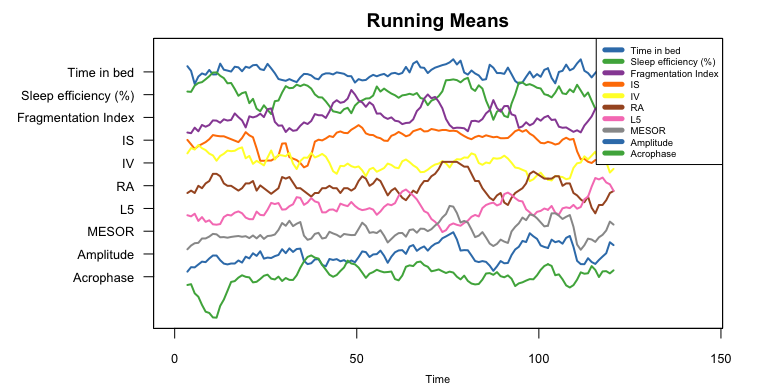 |
| 9 | NA | 119 | ~~27~~, 47 | 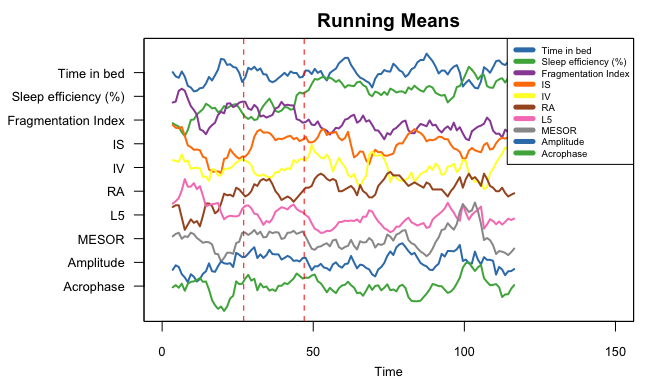 |
| 10 | NA | 122 | *87* | 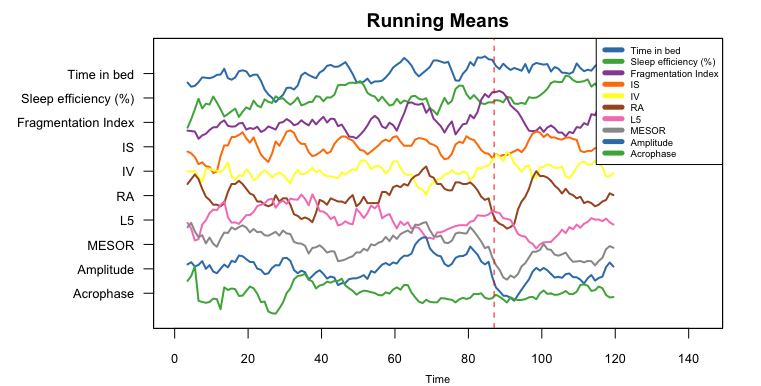 |
| 11 | NA | 123 | ~~48~~ | 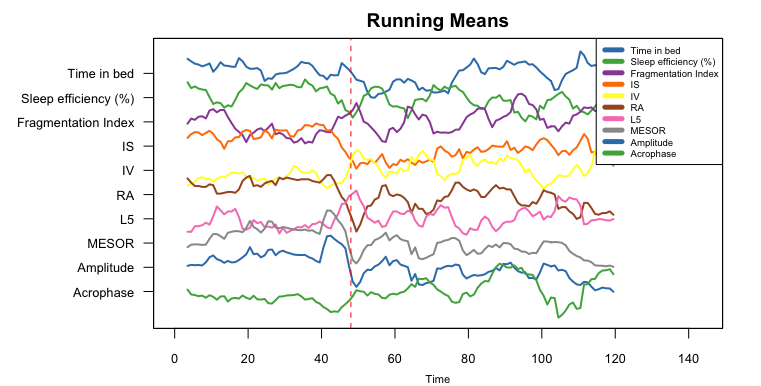 |
| 12 | NA | 117 | ~~33, 81~~ | 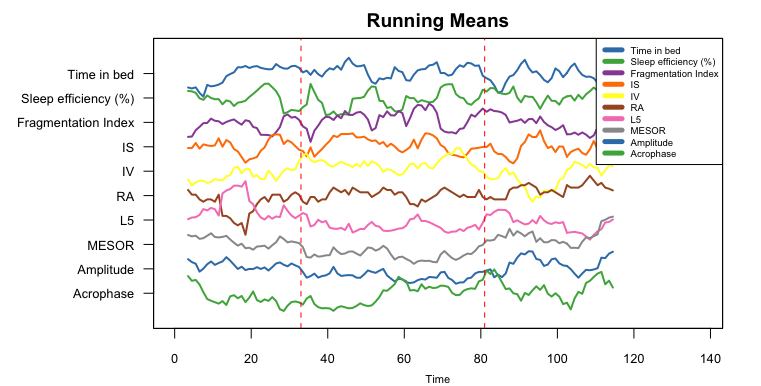 |
| 13 | NA | 123 | *60*, *78,* ~~97~~ | 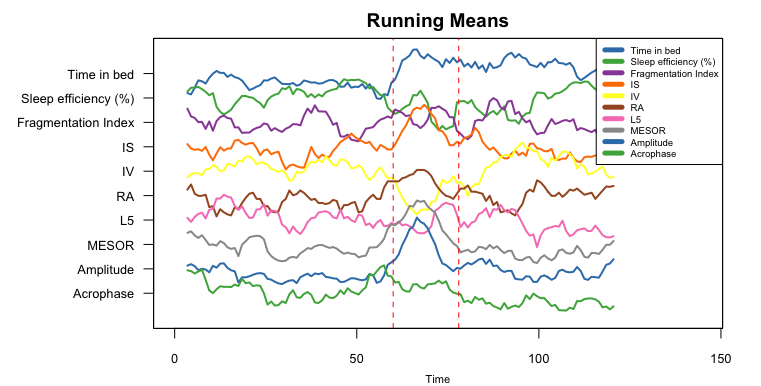 |
| 14 | NA | 122 | - | 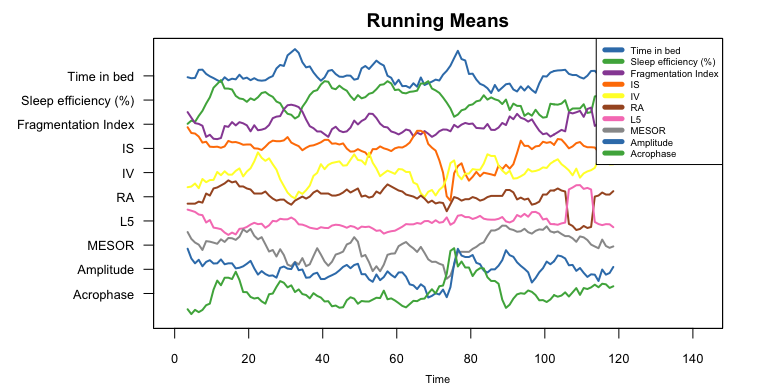 |
| **Excluded participants** (too short recording (less than 90 days) or transition before or after actigraphy recording) | | | | |
| 1 | NA | 43 | - | Too many missings |
| 2 | NA | 41 | - | 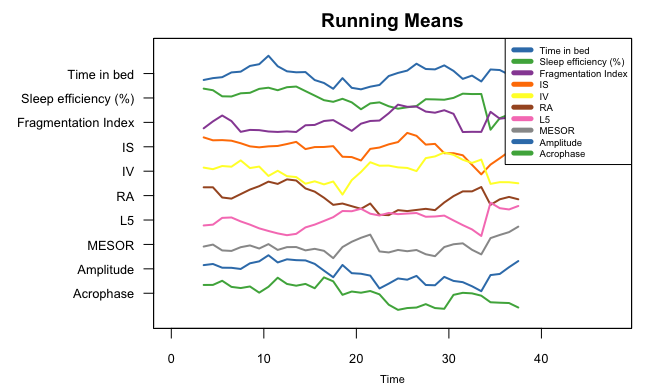 |
| 3 | NA | 51 | - | 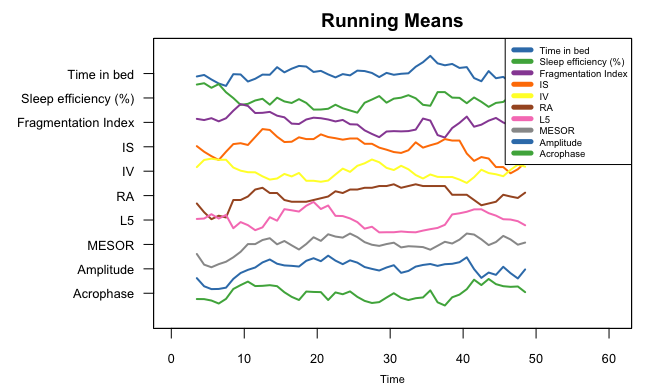 |
| 4 | NA | 54 | - | Too many missings |
| 5 | NA | 72 | - | Too many missings |
| 6 | 4-10 | 76 | - | 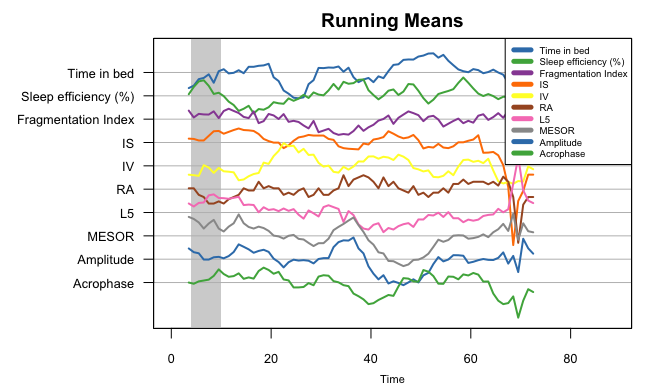 |
| 7 | 12-18 | 33 | - | 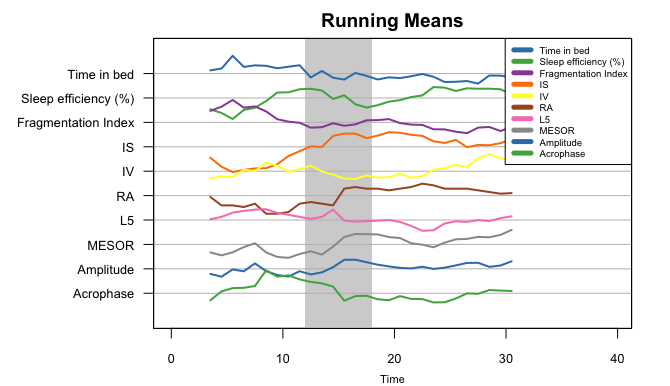 |
| 8 | 64-70 | 67 | ~~35~~ | 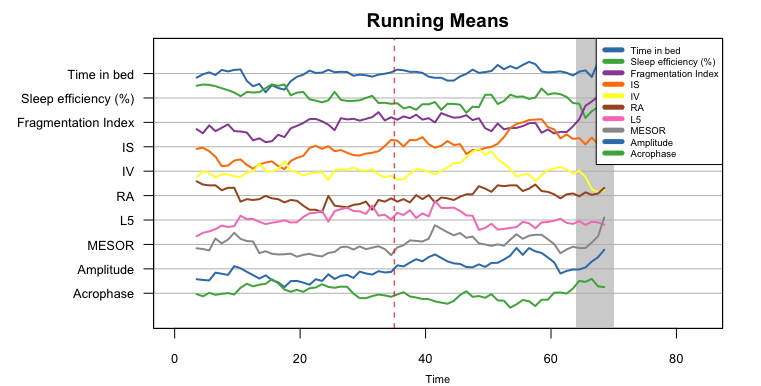 |
| 9 | transition after actigraphy end | 89 | 37, *58* | 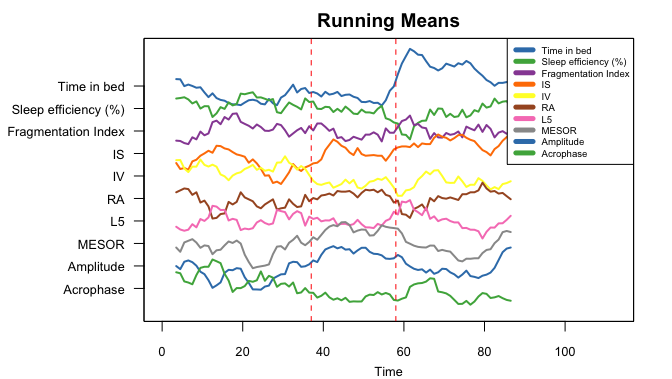 |
| 10 | transition before actigraphy start | 46 | - | 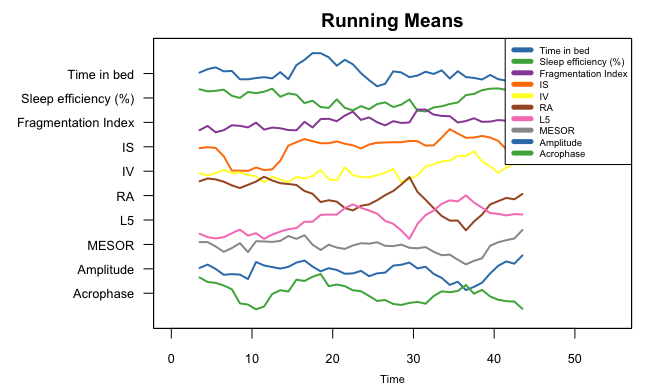 |
| 11 | transition after actigraphy end | 104 | *51* | 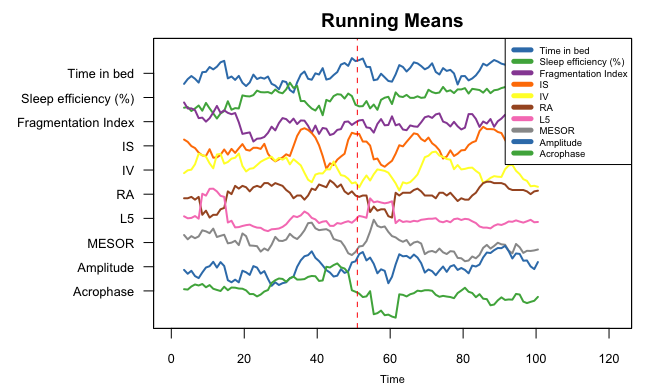 |
| 12 | transition after actigraphy end | 123 | ~~63~~, ~~80~~ | 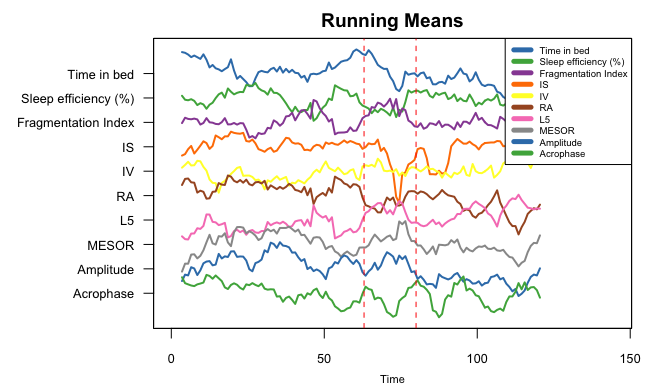 |
| 13 | transition after actigraphy end | 106 | ~~45~~ | 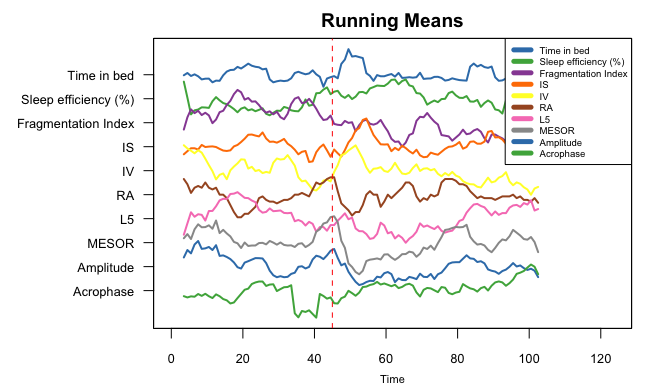 |
| 14 | transition after actigraphy end | 120 | - | 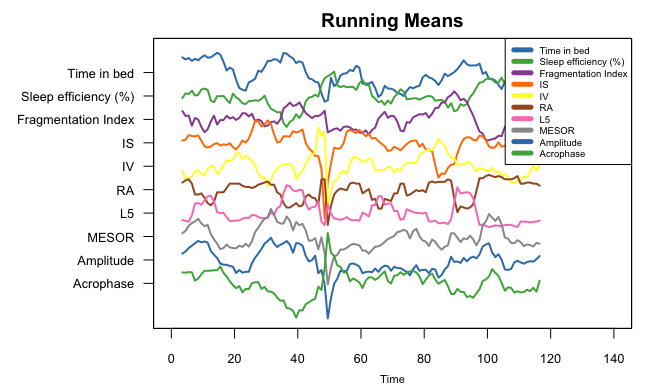 |
| 15 | transition after actigraphy end | 118 | ~~42~~, ~~82~~ | 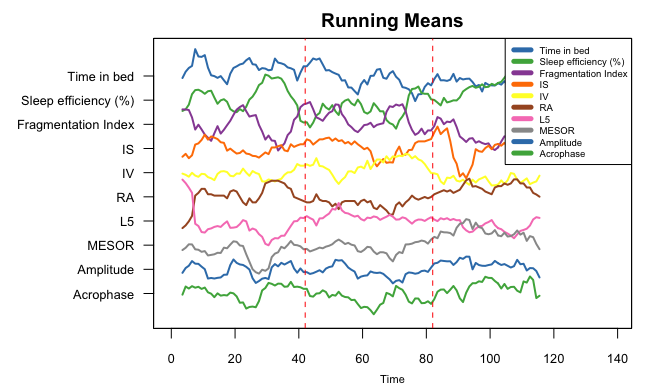 |
| 16 | transition after actigraphy end | 121 | ~~34~~, *98* | 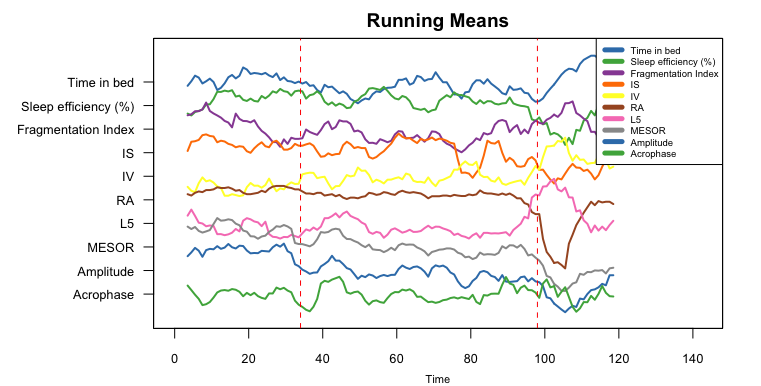 |
