# Supplementary material for: Individual-specific change points in circadian rest-activity rhythm and sleep in individuals tapering their antidepressant medication: an actigraphy study
Source: Sci Rep. 2024 Jan 9;14:855. doi: 10.1038/s41598-023-50960-1 (PMC10776866; doi:10.1038/s41598-023-50960-1)
Supplement: Supplementary file 5 — Supplementary Information 5. [file 41598_2023_50960_MOESM5_ESM.docx]

**Supplementary Figure S1**


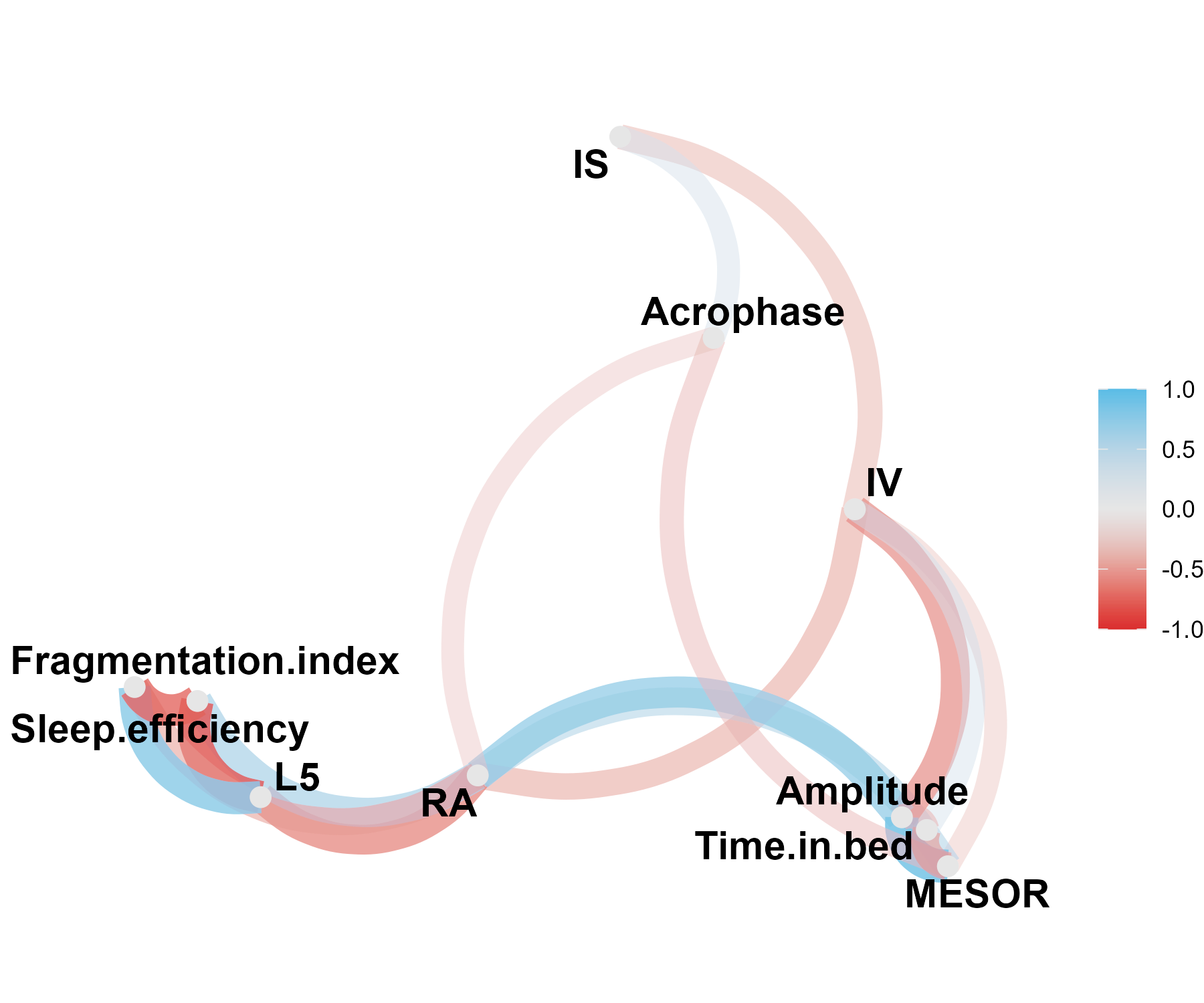


**Figure S1. A network plot indicating the strength of the correlations between RAR, physical activity, and sleep variables**

In Figure S1, the correlations between the following variables are visualized in a network; IS, IV, RA, L5, MESOR, Amplitude, Acrophase, Time in bed, Sleep Efficiency, and Fragmentation index. The blue lines represent positive correlations, and the red lines negative correlations. The strength of the correlation is visualized by the grading of the blue/red color. Only correlations above 0.3 were visualized. Networks were calculated by the R-package *corrr* 0.4.4^1^.

Reference

1. Kuhn M, Jackson S, Cimentada J (2022). *corrr: Correlations in R*. https://github.com/tidymodels/corrr, https://corrr.tidymodels.org.
